# (−)-6-epi-Artemisinin, a Natural Stereoisomer of (+)-Artemisinin in the Opposite Enantiomeric Series, from the Endemic Madagascar Plant *Saldinia proboscidea*, an Atypical Source

**DOI:** 10.3390/molecules26185540

**Published:** 2021-09-12

**Authors:** Saholinirina Randrianarivo, Claudine Rasolohery, Sitraka Rafanomezantsoa, Heriniaina Randriamampionona, Liti Haramaty, Roger Marie Rafanomezantsoa, Eric H. Andrianasolo

**Affiliations:** 1 Ecole Doctorale de Géochimie et Chimie Médicinale (GEOCHIMED), Université de Fianarantsoa, Fianarantsoa 301, Madagascar; mrhortensia@gmail.com (S.R.); carasolohery@gmail.com (C.R.); mitombinasitraka@gmail.com (S.R.); heriniaina.andri@gmail.com (H.R.); rmrafano@gmail.com (R.M.R.); 2 DMCS, Rutgers, The State University of New Jersey, New Brunswick, NJ 08901, USA; haramaty@marine.rutgers.edu; 3 Departement Chimie, Centre National de Recherches Industrielle et Technologique (CNRIT), Antananarivo 101, Madagascar; 4 Department of Biology, University of Waterloo, Waterloo, ON N2L 3G1, Canada

**Keywords:** *Saldinia proboscidea*, *Artemisia* species, antimalaria, antiproliferative, SARS-CoV-2 inhibitor

## Abstract

Chemical and biological investigation of the Madagascar endemic plant *Saldinia proboscidea* led to the isolation of an isomer of artemisinin, (−)-6-epi-artemisinin (**2**). Its structure was elucidated using a combination of NMR and mass spectrometry. The absolute configuration was established by chemical syntheses of compound **2** as well as a new stereoisomer (**3**). The comparable bioactivities of artemisinin (**1**) and its isomer (−)-6-epi-artemisinin (**2**) revealed that this change in configuration was not critical to their biological properties. Bioactivity was assessed using an apoptosis induction assay, a SARS-CoV-2 inhibitor assay, and a haematin polymerization inhibitory activity (HPIA) assay. This is the first report of an artemisinin-related compound from a genus not belonging to *Artemisia* and it is the first isolation of an artemisinin-related natural product that is the opposite enantiomeric series relative to artemisinin from *Artemisia annua*.

## 1. Introduction

Artemisinins are one of the most important antimalarial drug classes in use today [1]. However, the production of these drugs is expensive compared to previous antimalaria drugs. A number of studies have been done to enhance the biosynthetic pathway of artemisinin and its expression in different organisms, such as *E. coli* and *S. cerevisiae* [2]. Alternative methods for producing artemisinins have been studied, for example, by improving the yield of the precursor, artemisinic acid, in genetically engineered yeast [1,2]. Nevertheless, this semi-synthetic method has also been found to be expensive because of the cost of growth media and culture maintenance. Artemisinin was originally isolated from the sweet wormwood plant *Artemisia annua*. The biosynthesis of artemisinin (**1**) has been reviewed and generally accepted to be from terpene [3]. Three phases constitute the biosynthesis of **1**. Phase one is the conversion of isopentenyl pyrophosphate (IPP) and its isomer dimethylallyl pyrophosphate (DMAPP) to amorpha-4,11-diene. Phase two is the modification to the isopropylidene group (C-11, C-12 and C-13) in amorpha-4,11-diene, yielding putative biosynthetic intermediates such as dihydroartemisinic acid. Phase three is the conversion from dihydroartemisinic acid to artemisinin (**1**). Artemisinins also have a remarkable ability to kill cancer cells, and recently these drugs have been repositioned to be used as potential antiproliferatives [4]. The major mechanism of action of artemisinin appears to be due to toxic-free radicals generated by its endoperoxide moiety, and the ensuing cell cycle arrest, induction of apoptosis and inhibition of tumor angiogenesis [5]. Recently, artemisinin was found to have an inhibitory effect on SARS-CoV-2, with half of the maximal effective concentration EC_50_ = 64 μM [6].

**Figure 1:**
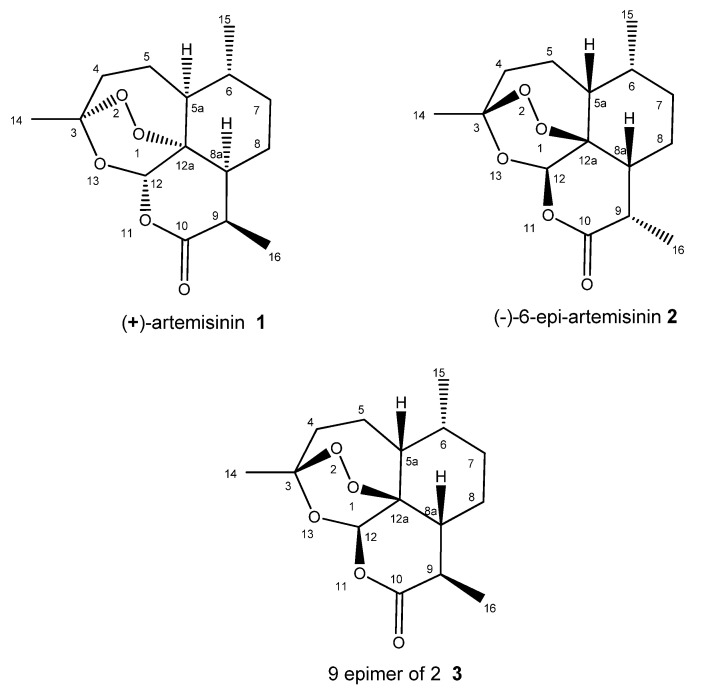
(+)Artemisinin (**1**), (−)-6-epi-artemisinin (**2**) and 9 epimer of **2** (**3**).

In our ongoing efforts to discover and develop new natural biomedicines, especially antiproliferative drugs, we have screened extracts from several terrestrial organisms and have found that those isolated from the Madagascar endemic plant species *Saldinia proboscidea*, which is used for the treatment of malaria by the indigenous people of Madagascar, possessed a striking ability to induce apoptosis in cancer cell lines. Subsequently, extracts were subjected to bioassay-guided purification that resulted in the isolation of a new stereoisomer of artemisinin (**2**) (Figure 1).

## 2. Results

### 2.1. Extraction and Structure Elucidation

Leaves of the Madagascar endemic plant *Saldinia proboscidea* were collected, dried and ground into a fine powder. A voucher specimen is available from the “Ecole doctorale de Géochimie et Chimie Médicinale (GEOCHIMED)”, University of Fianarantsoa, as collection number MG-UF-GEOCHIMED 01-2016. The green colored plant powder was stored at 4 °C until subsequent chemical extraction. A total of 490 g of the green plant powder was macerated in 2 L of ethanol for 3 days. The solid residue was separated from the liquid extract by filtration. After evaporation, the dried extract (70 g) was fractionated by solvent partition between water and ethyl acetate. The ethyl acetate fraction (7.5 g) was subsequently fractionated over a solid phase extraction cartridge (SPE) using the following solvent system: hexane, hexane-ethyl acetate, ethyl acetate-methanol and methanol; four sub-fractions were obtained. The ethyl acetate-methanol fraction was found to be active in the apoptosis induction assay, and it was then subjected to HPLC to give 2.5 mg of compound **2** for a bioassay, NMR and MS purposes.

**Figure 2:**
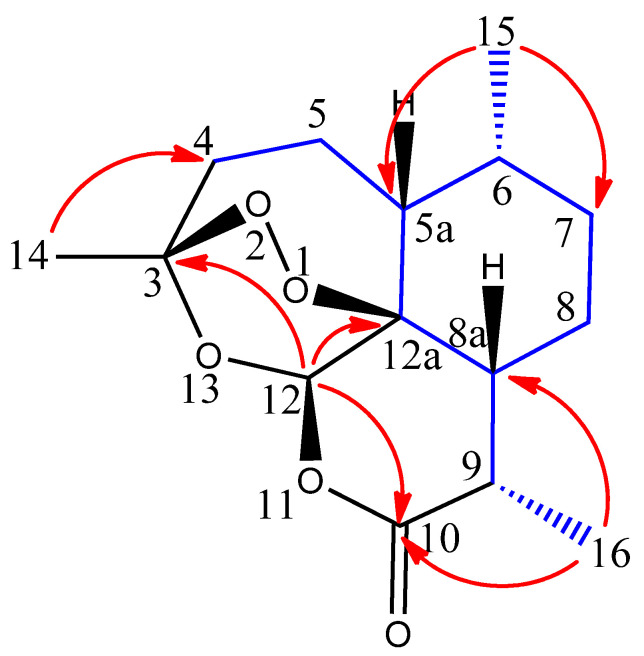
Key COSY (blue) and HMBC (red) correlations for (−)-6-epi-artemisinin (**2**).

The molecular formula of compound **2** was established as C_15_H_22_O_5_ on the basis of HRESIMS [*m/z* 283.1547 (M + H)^+^ (calculated for C_15_H_23_O_5_, 283.1540)]. This indicated that it possessed the exact molecular formula of the known compound artemisinin (**1**). The ^1^H NMR of compound **2** clearly displayed three methyl groups at δ_H_ 1.46 (s), δ_H_ 1.01 (d, 5.96 Hz) and δ_H_ 1.22 (d, 7.28 Hz). Another interesting signal was located at δ_H_ 5.89 (s), which is found to be very similar to that of a proton shift in compound **1** at δ_H_ 5.87 (s). These signals are characteristic of protons belonging to a methine group that is connected to two oxygen atoms (e.g., C-12). Furthermore, the presence of a proton at δ_H_ 3.41 (dq, 7.2, 5.3) is similar to that found in (**1**) at δ_H_ 3.40 (dq, 7.2, 5.4). These signals are characteristic of protons adjacent to a carbonyl group (e.g., C-9). The remaining proton signals are from methine and methylene groups ranging from δ_H_ 1.10 to 2.41. The ^13^C NMR of compound **2** revealed the presence of fifteen carbon signals at δ_C_ 13.9, 21.2, 24.8, 26.2, 26.6, 34.3, 35.0, 37.3, 38.9, 46.4, 51.4, 80.9, 95.1, 106.8 and 173.4. Multiplicity edited HSQC revealed that three carbons did not show correlation to any protons, clearly indicating that compound **2** had three quaternary carbons, one of which was the carbonyl group at δ_C_ 173.4. The other two quaternary carbon signals at δ_C_ 80.9 and δ_C_ 106.8 were attributed to the carbons connected to one oxygen atom and two oxygen atoms, respectively. To assign the remaining carbon signals, and to differentiate methine (CH) or methyl (CH_3_) signals from methylene (CH_2_) signals, a multiplicity edited HSQC was performed. The following carbon signals were found to belong to methyl groups: δ_C_ 13.9, 21.2 and 26.2, with their corresponding proton signals at δ_H_ 1.22 (d, 7.28 Hz), 1.01 (d, 5.96 Hz) and 1.46 (s), respectively. The following carbon signals were found to belong to methine groups: δ_C_ 35.0, 38.9, 46.4, 51.4 and 95.1. The corresponding proton signals were located at δ_H_ 3.41 (dq, 7.2, 5.3), 1.42 (m), 1.80 (m), 1.38 (m) and 5.89 (s), respectively. Finally, the carbon signals at δ_C_ 24.8, 26.6, 34.3 and 37.3 were found to belong to methylene groups, and their corresponding proton signals were found at δ_H_ 1.10 (m), 1.90 (m), δ_H_ 2.00 (m), 2.00 (m), δ_H_ 1.10 (m), 1.81 (m), δ_H_ 2.09 (m), 2.41 (m). To build the structure of compound **2**, several partial structures were established using HMBC correlations. For example, a key correlation was found between the proton at δ_H_ 5.89 (s) and the carbonyl group δ_C_ 173.4, as well as two other carbons at δ_C_ 80.9 and δ_C_ 106.8. These correlations are strikingly similar to those found in the structure of artemisinin (**1**). Given that HMBC correlations are relatively strong from methyl group signals, other partial structures built from these correlations were as follows: a correlation between the proton δ_H_ 1.46 (s) and the carbons at δ_C_ 37.3 and δ_C_ 106.8; a correlation between the proton δ_H_ 1.01 (d, 5.96 Hz) and the carbons at δ_C_ 51.4, 38.9 and 34.3; finally, a correlation between the proton δ_H_ 1.22 (d, 7.28 Hz) and the carbons δ_C_ 35.0, 46.4 and the carbonyl group δ_C_ 173.4. To connect all these partial structures, a combination of COSY and HMBC correlations were used (Figure 2), as well as a comparison to the structure of artemisinin (**1**). The complete carbon and proton shifts for compound **2** are displayed in Table 1. From all the above, and given the same molecular weight and molecular formula of **1** and **2**, and the close comparability of their ^1^H and ^13^C NMR data, it was concluded that compound **2** was a stereoisomer of artemisinin (**1**).

### 2.2. Relative Configuration of (−)-6-epi-Artemisinin (***2***) by ROESY Data Analyses

**Figure 3:**
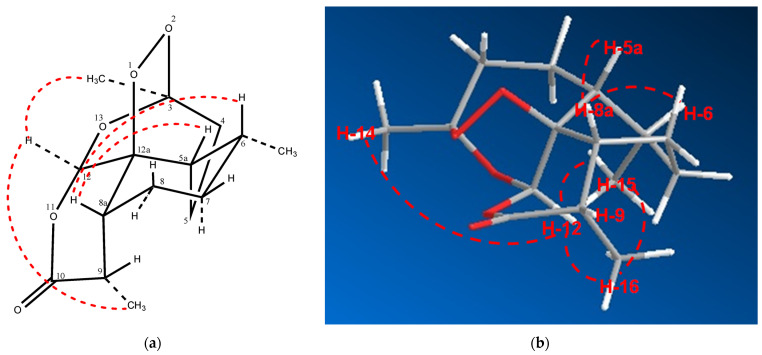
Selected ROESY cross peaks for (−)-6-epi-artemisinin (**2**): (**a**) 3D structure drawing; (**b**) 3D model by chemBioDraw Ultra 12.0.

Similar to artemisinin (**1**), compound **2** has seven stereocenters: C-3, C-5a, C-6, C-8a, C-9, C-12 and C-12a. The absolute configuration of artemisinin (**1**) was established by X-ray crystallography; however, given the small amount of material isolated in this study, a crystal structure of compound **2** was ruled out. The relative configuration of **2** was established on the basis of ROESY data analyses. Moreover, the ^13^C NMR data of compounds **2** and (−)-artemisinin are strikingly similar, and they both have similar values of negative optical rotation: -58.1 and -69.5, respectively. On the other hand, the sign of the optical rotation of artemisinin (**1**) is positive at +87.94. The relative configuration of **2** was found to be very similar to that found for (−)-artemisinin, and ROESY cross peaks between the following protons confirmed these assignments: H-5a and H-8a, H-12 and H-14 and H-8a and H-9 (Figure 3). However, the configuration at C-6 is different from (−) -artemisinin but similar to **1.** If the configuration at C-6 was similar to that found in (−)-artemisinin, we should see a ROESY cross peak between H-6 and H-12. However, we observed a ROESY cross peak between H-6 and H-8a as well as H-15 and H-12, suggesting that C-6 had the same configuration as that found in **1**. The relative configuration of **2** was then confirmed, and its structure was established as shown.

### 2.3. Absolute Configuration of (−)-6-epi-Artemisinin (***2***) by Chemical Syntheses

**Figure 4:**
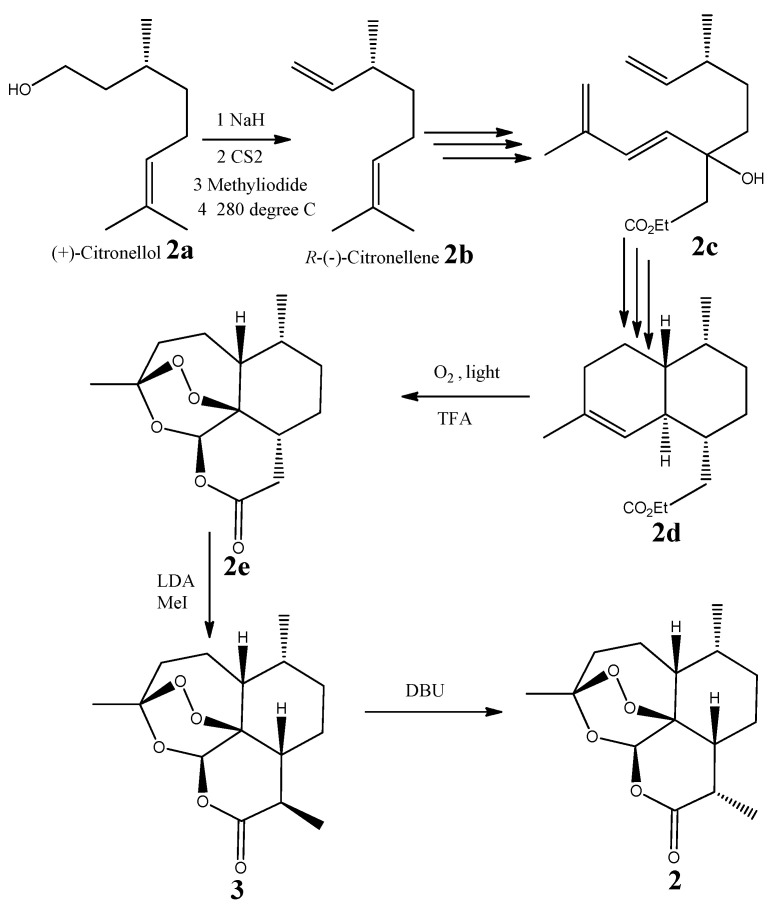
Chemical syntheses of compound (**3**) (epimer of compound (**2**) at C-9) and (−)-6-epi-artemisinin (**2**).

The absolute configuration of compound **2** was established by direct chemical synthesis of (−)-6-epi-artemisinin (**2**). An efficient chemical synthesis of (−) artemisinin was developed by Krieger et al. [7], and we chose to follow the same chemical synthesis route by a different starting material. We started with commercially available (+)-citronellol (**2a**), which is of known configuration. We converted this starting material into (*R*)-(−)-3,7-dimethyl-1,6-octadiene or *R*-(−)-citronellene (**2b**) ([α]^22.5^_D_ −10 (*c* 0.5, CHCl_3_), and then used several steps from the Krieger synthesis to successively produce ethyl (6*R*)-3-hydroxy-6-methyl-3-((E)-3-methylbuta-1,3-dien-1-yl)oct-7-enoate (**2c**), ethyl 2-((1*R*,4*R*,4a*R*,8a*R*)-4,7-dimethyl-1,2,3,4,4a,5,6,8a-octahydronaphthalen-1-yl)acetate (**2d**) and (3*S*,5a*R*,6*R*,8a*R*,12*R*,12a*R*)-3,6-dimethyloctahydro-12*H*-3,12-epoxy [1,2] dioxepino [4,3-i]isochromen-10(3*H*)-one (**2e**) (Figure 4). This latter product was transformed to a new epimer (**3**) of **2** at C-9 by treatment of **2e** with lithium diisopropylamide (LDA) and methyl iodide (MeI). The final step of the route was to convert compound **3** into the final product, (−)-6-epi-artemisinin (**2**), with the addition of 1,8-diazabicyclo [5.4.0]undec-7-ene (DBU). The ^1^H and ^13^C NMR of the natural product and synthetic 6-epi-artemisinin (**2**) were compared and found to be identical. This was additionally confirmed by mixing 1 mg of the synthetic product **2** with 1 mg of the natural product **2** and observing an identically overlapped ^1^H and ^13^C NMR spectra. The absolute configuration of **2** was established as 3*S*,5a*R*,6*R*,8a*R*,9*S*,12*R*,12a*S* from its opposite sign of optical rotation compared to artemisinin (**1**), and its identical optical rotation to synthetic **2,** [α]^22.5^_D_ −58.2 (*c* 0.9, CHCl_3_) 

### 2.4. Molecular Docking of (−)-6-epi-Artemisinin (***2***) with the RBM of Spike Protein of SARS-CoV-2

**Figure 5:**
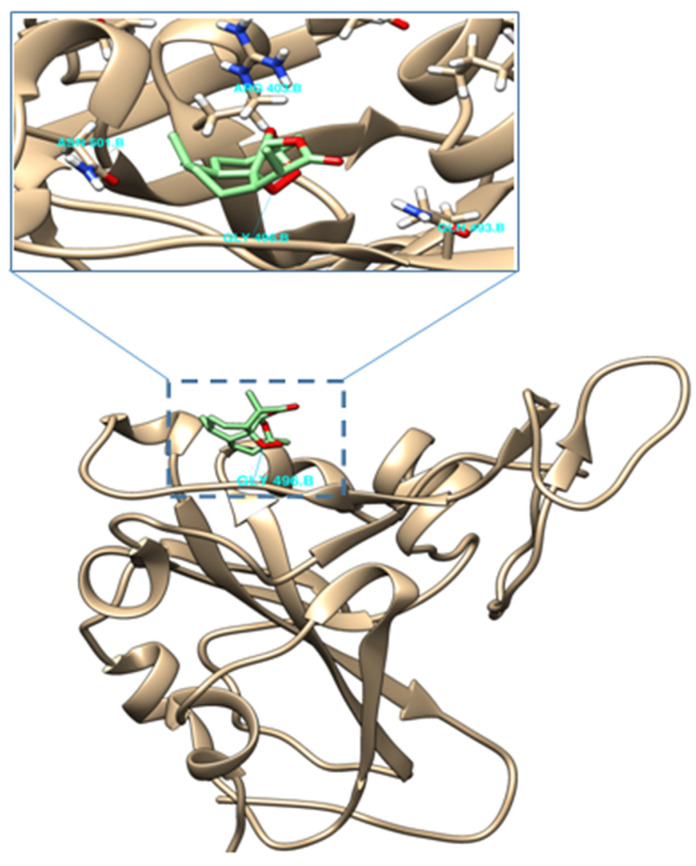
Interaction of (−)-6-epi-artemisinin (**2**) with the RBM of the spike protein of SARS-CoV-2 (Lys-353 binding hotspot).

Molecular docking studies of (−)-6-epi-artemisinin (**2**) demonstrated the presence of a good interaction with the Lys-353 binding hotspot, situated in the receptor-binding motif (RBM), which in turn is present in the receptor binding domain (RBD) of the spike protein (Figure 5). The obtained binding energy (Vina score) in this area was −6.2 kcal/mol, compared to −6.5 kcal/mol for artemisinin [8]. We also notice the presence of hydrogen bonding between one of the oxygens in the peroxy bridge and Gly 496 residue; it is worth noting that such a type of hydrogen bonding was not present in the case of artemisinin. In contrast, molecular docking of this isomer did not show any type of interactions with the Lys-31 binding hotspot of the RBM; this is different to artemisinin which was previously [8] shown to interact with this region of the RBM, albeit at a slightly lower binding energy, 5.6 kcal/mol.

### 2.5. Biological Activities

#### 2.5.1. Apoptosis Induction

**Figure 6:**
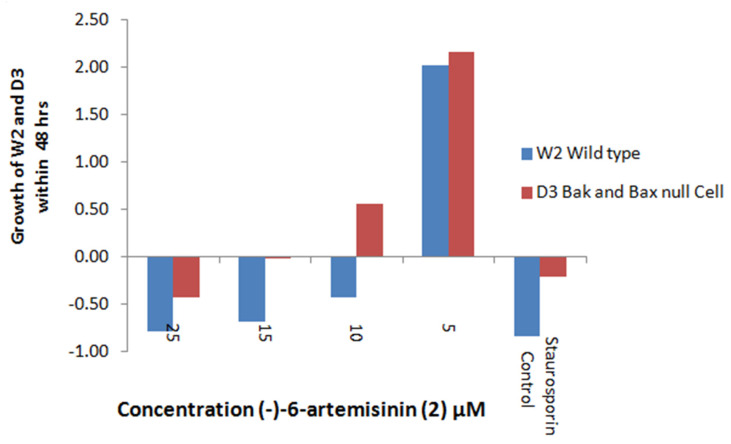
Induction of apoptosis of (−)-6-epi-artemisinin (**2**) with IC_50_ of 11 ± 0.71 μM.

**Figure 7:**
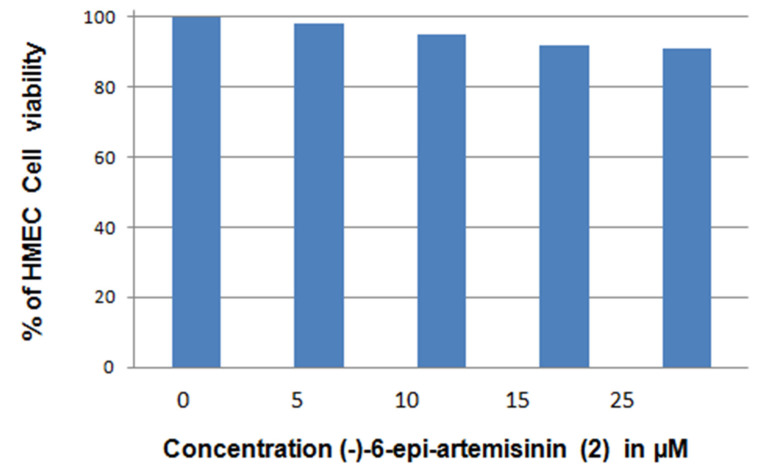
Effect of (−)-6-epi-artemisinin (2) on cell viability of normal Human Mammary Epithelial Cells HMEC.

The antimalarial activity of (+)-artemisinin (**1**) and its synthetic antipode (−) artemisinin was recently assessed and was found to be not stereospecific [7]. However, the antiproliferative activity of different artemisinin isomers has never been studied in the past. Artemisinin (**1**) shows antiproliferative activity to MCF breast cancer cells with an IC_50_ of 9.13 ± 0.07 μM. [9], whereas dihydroartemisinin has an IC_50_ of 5.27 ± 0.01 μM in these same cells [10]. To address this subject, we performed an apoptosis induction assay with compound **2** in order to evaluate the effect of the change in configurations on this activity. To develop a cell-based assay specific for the identification of apoptosis-inducing compounds that are potential antiproliferative agents, we used two genetically engineered mouse epithitial cell lines: W2 apoptotic competent and D3 apoptotic deficient. Compounds that have the capacity to kill W2 and not D3 cells are consistent with proapoptotic, potentially antiproliferative activity [11]. Compounds that meet these criteria activate apoptosis upstream in a pathway that requires Bax and Bak. Moreover, compounds that indiscriminately kill both apoptosis-competent W2 and apoptosis-defective D3 cells can be used to eliminate those that are non-specifically toxic [12]. The chemical (−)-6-epi-artemisinin (**2**) induces apoptosis with IC_50_ values of 10 μM and 11 ± 0.71μM (Figure 6); (−)-6-epi-artemisinin (**2**) does not have a sizeable effect on the viability of normal Human Mammary Epithelial Cells HMEC (Figure 7).

#### 2.5.2. SARS-CoV-2 Inhibition

**Figure 8:**
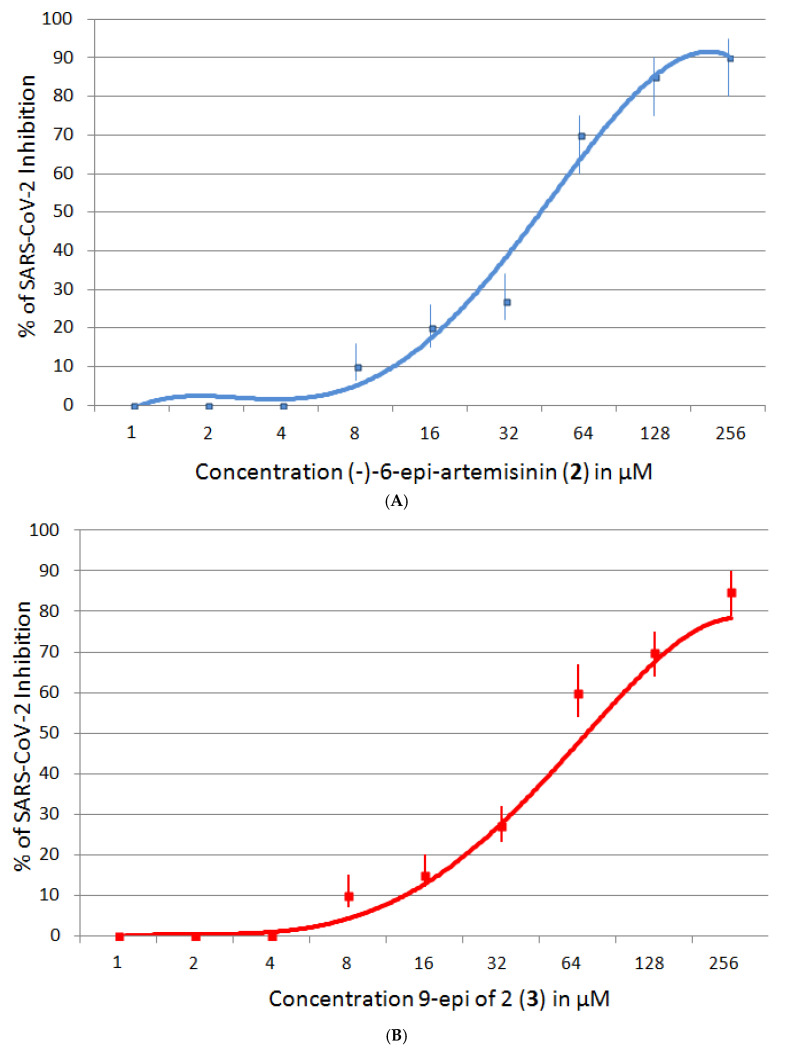
SARS-CoV-2 Inhibitory assay of (−)-6-epi-artemisinin (**2**) (**A**) with EC_50_ = 49 *±* 0.32 μM and SARS-CoV-2 inhibitory assay of 9 epimer of 2 (**3**) (**B**) with EC_50_ = 54 *±* 0.27 μM.

**Figure 9:**
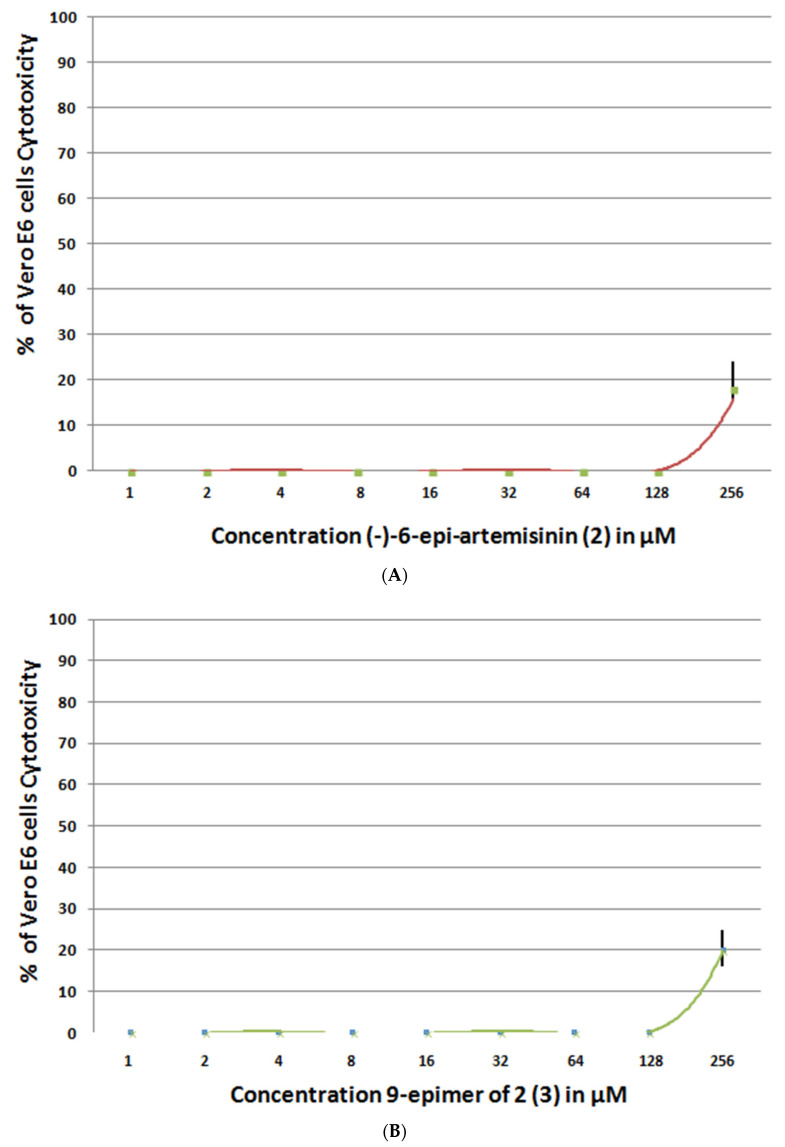
The percentage of cytotoxicity of (−)-6-epi-artemisinin (**2**) against Vero E6 cells with (**A**) CC_50_ > 250 μM and 9 epimer of 2 (**3**) with (**B**) CC_50_ > 200 μM.

To evaluate the anti-SARS-CoV-2 activity of these new artemisinin analogs, we used the protocol developed by Ruiyuan et al. [6], in which Vero E6 cells were treated with a SARS-CoV-2 strain. The cytopathic effects of the virus were assessed by measuring the viability of Vero E6 cells after treatment with the virus. Artemisinin (**1**) and (−)-6-epi-artemisinin (**2**) inhibited the growth of SARS-CoV-2. Based on these results, we conclude that the activities are not dependent on the change in configurations. Moreover, the configuration of the C-9 methyl group was not critical to their biological properties, since (−)-6-epi-artemisinin (**2**) and 9 epimer of **2** (**3**) inhibited the growth of SARS-CoV-2 with comparable bioactivities (Figure 8). The chemical (−)-6-epi-artemisinin (**2**) and 9 epimer of **2** (**3**) do not have a significant effect on the viability of Vero E6 cells CC_50_ > 200 µM for either compound (Figure 9).

#### 2.5.3. Antimalaria Assay

**Figure 10:**
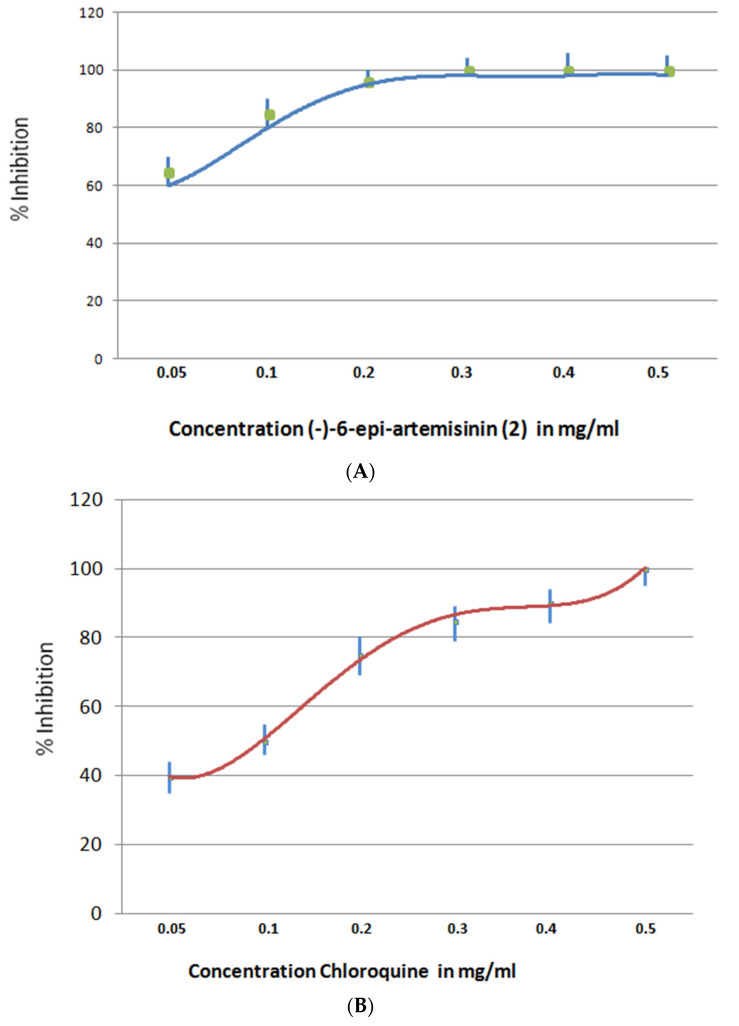
Inhibition of haem polymerization formation by (**A**) (−)-6-epi-artemisinin (**2**) (blue) with IC_50_ = 3.5 ± 0.002 nM and (**B**) chloroquine (red) with IC_50_ = 18 *±* 0001 nM.

Antimalarial activity was tested to (−)-6-epi-artemisinin (**2**) based on inhibition of haem polymerization [13]. Haem is a by-product of haemoglobin digestion and is used by the parasite as a source of most of its essential amino acids. It is potentially toxic to biological membranes and parasite enzymes, and it is thus sequestered in the form of an insoluble crystalline polymer, haemozoin or malaria pigment. Identifying molecules that interfere with haem polymerization is the basic principle of the assay. Haem can be polymerized in vitro, in the absence of proteins, from a solution of haematin at 70 °C or 37 °C at acidic pH. In vitro quantification of haem polymerization allows the identification of molecules with haem polymerization inhibitory activity (HPIA) and the assessment of their HPIA, relative to that of standard antimalarials like chloroquine. The results showed that (−)-6-epi-artemisinin (**2**) inhibits haem polymerization formation with IC_50_ = 3.5 ± 0.002 nM, compared to that of chloroquine IC_50_ = 18 ± 0.001 nM (Figure 10).

## 3. Discussion

To date, the only known sources of artemisinin (**1**) have been species of the genus *Artemisia,* which comprises some 500 species [14], the best-known being *A. annua, A. scoparia,* and *A. paviflora*. Given the medicinal importance of **1**, there is a continuing need to search for other alternatives for its production. To our knowledge, this is the first time that an isomer of **1** was isolated from a plant species, namely *Saldinia proboscidea,* that does not belong to the genus *Artemisia*. This finding widens the search for new antimalarial and antiproliferative drugs related to artemisinin (**1**) in species not belonging to the genus *Artemisia*. *Saldinia* and *Artemisia* are in different taxonomic orders; *Saldinia* is in *Gentianales* and *Artemisia* is in *Asterales*, which indicates that they are not closely related botanically (Figure S14 Supplementary Material). The isolation of **2** from *Saldinia proboscidea*, an artemisinin-related natural product that is the opposite enantiomeric series relative to artemisinin from *Artemisia annua*, implies that a synthase in *Saldinia proboscidea* forms an amorphadiene stereoisomer that has opposite configurations to that found in *Artemesia annua* at all stereocenters except at C-6.

Apoptosis, the naturally programmed cell death, when activated is a natural way to combat any type of cellular damage and malfunctioning. The vast majority of human solid tumors are of epithelial origin, and defects in apoptosis, mostly upstream of Bax and Bak, play important roles in both tumor suppression and mediation of chemotherapeutic response [11]. Apoptosis is hallmarked by the formation of apoptotic bodies, and it is associated with alterations in Bax and Bcl-2 protein expressions. Cancer cells do not have the ability to activate this process. Since (−)-6-epi-artemisinin (**2**) selectively induces apoptosis by acting solely to cancer cells, as demonstrated by the killing of epithelial cancer cells W2 but not normal Human Mammary Epithelial Cells (HMEC), (−)-6-epi-artemisinin (**2**) induces apoptosis upstream of Bax and Bak, and it may have potential for use as an antiproliferative agent that exploits the apoptosis pathway in tumor cells.

The COVID-19 pandemic caused by severe acute respiratory coronavirus 2 (SARS-CoV-2) has killed more than 4 million people since December 2019. The discovery of drug candidates with anti-SARS-CoV-2 potential is needed to supply antiviral drug research for COVID-19. Molecular docking analyses displayed that (−)-6-epi-artemisinin (**2**) interacts with the RBM of Spike protein of SARS-CoV-2. The results revealed that (−)-6-epi-artemisinin (**2**) and 9 epimer of **2** (**3**) inhibited the growth of SARS-CoV-2, and, consequently, they are promising drug candidate leads for anti-SARS-CoV-2 drug research and development.

It is also demonstrated that (−)-6-epi-artemisinin (**2**) is able to interfere with the haem polymerization at a very low concentration. The mechanism of action of artemisinins in killing malaria parasite is believed to involve radical reaction and damage of parasite lipids and proteins. In addition to that, artemisinin can prevent the formation of malaria pigment by irreversibly inhibiting heme crystallization. Our results confirmed that (−)-6-epi-artemisinin (**2**), an artemisinin analogue, inhibits heme crystallization. This will have an impact on how to develop new artemisinin derivatives for combatting growing resistance of P. falciparum to artemisinin.

The endoperoxide moiety of artemisinin and its analogues is the critical part of these molecules. The changes in configuration do not have enormous effect on the bioactivities of these molecules. This conclusion was confirmed by all biological assays in this present investigation. Furthermore, this moiety is the source of radical reaction and the most reactive site of these molecules. As an example, in the antimalaria activity of these molecules, the two oxygens present in the peroxide group with their electron density is an attractive site for the iron positively charged in heme. Ma et al. [15] had demonstrated that heme–drug adducts, produced after the radical activation of artemisinins, can inhibit β-hematin crystallization and heme detoxification, a pathway which complements the deleterious effect of radicals generated via parent drug activation.

## 4. Materials and Methods

### 4.1. General Experimental Procedures

Optical rotations were measured on JASCO P 1010 polarimeter; UV and FT-IR spectra were obtained by employing Hewlett Packard 8452A and Nicolet 510 instruments, respectively. All NMR spectra were recorded on a Bruker Avance DPX400 spectrometer. Spectra were referenced to the residual solvent signal with resonances at δ_H/C_ 7.26/77.1 (CDCl_3_). ESI MS data were acquired on a Waters Micromass LCT Classic mass spectrometer and Varian 500-MS LC Ion Trap. HPLC separations were performed using Waters 510 HPLC pumps, a Waters 717 plus autosampler, and Waters 996 photodiode array detector. All solvents were purchased as HPLC grade.

### 4.2. Extraction and Isolation Procedures

Extraction was performed by the maceration of 490 g of the green plant powder with 2 L of ethanol for 3 days, then separation by filtration, liquid filter bag polyester multifilament mesh 100 micron of the solid residue and the liquid extract. Evaporation was conducted by rotavapor and acquisition of the dried extract (70 g). Fractionation was performed by solvent partition between water and ethyl acetate. The ethyl acetate fraction (7.5 g) was subsequently fractionated over a solid phase extraction cartridge (SPE) using the following solvent system: hexane, hexane-ethyl acetate, ethyl acetate-methanol and methanol, and four sub-fractions were obtained. The ethyl acetate–methanol fraction was found to be active in the apoptosis induction assay, and it was then subjected to HPLC to give 2.5 mg of compound 2 for bioassay, NMR and MS purposes.

HPLC Stationary Phase Optimization. Purification of the artemisinin analog was performed using HPLC as described above. The best isolation was achieved on columns with aromatic groups bonded to the stationary phase. The best columns were Luna 5μ C18 250 × 4.6 mm (Phenomenex) and Betasil C18 5 μm 250 × 4.6 mm (Thermo Fisher Scientific, Waltham, MA, USA), which give 2.5 mg of pure (−)-6-epi-artemisinin (**2**) at *t_R_* = 9 min.

**(−)-6-epi-artemisinin (2)**: white powder. [α]^22.5^_D_ −58.1 (c 0.9, CHCl_3_); IR νmax 2953, 2931, 2911, 2850, 1740, 1454, 1383, 1279, 1235, 1199, 1183, 1152, 1117, 1034, 1011, 995, 883, 840, 794, 758 cm^−1^; 1H NMR and 13C NMR, see Table 1; HRESIMS [*m/z* 283.1547 (M + H)^+^ (calculated for C_15_H_23_O_5_, 283.1540)].

**Synthetic (compound 2)**: white powder. [α]^22.5^_D_ −58.2 (c 0.9, CHCl_3_); IR νmax 2953, 2931, 2911, 2850, 1740, 1454, 1383, 1279, 1235, 1199, 1183, 1152, 1117, 1034, 1011, 995, 883, 840, 794, 758 cm^−1^, HRESIMS [*m/z* 283.1547 (M + H)^+^ (calculated for C_15_H_23_O_5_, 283.1540)]. ^1^H NMR and ^13^C NMR identical to Table 1.

**9 Epimer of 2 (compound 3)**: white powder [α]^22.5^_D_ −50.1 (c 0.9, CHCl_3_); IR νmax 2988, 2956, 2931, 2908, 2850, 1732, 1454, 1396, 1378, 1366, 1281, 1208, 1158, 1138, 1110, 1034, 1011, 996, 963, 929, 879, 863, 832, 758 cm^−1^; ^1^H NMR and ^13^C NMR, see Table S1 Supplementary Material; HRMS (*m/z*): (M+Na)^+^ (calculated for C_15_H_22_O_5_Na, 305.1365 found 305.1361).

**Compound 2b**: *R*(−)-Citronellene as a yellow liquid. [α]^22.5^_D_ −10 (c 0.5, CHCl_3_), ^1^H NMR (400 MHz, CDCl_3_) δ_H_ 5.70 (ddd, J = 17.5, 10.3, 7.5 Hz, 1 H), 5.13–5.07 (m, 1 H), 5.00–4.88 (m, 2 H), 2.19–2.05 (m, 1 H), 2.03–1.90 (m, 2 H), 1.68 (s, 3 H), 1.60 (s, 3 H), 1.37–1.26 (m, 2 H), 0.99 (s, 3 H); 13C (100 MHz, CDCl_3_) δ_C_ 144.9, 131.4, 124.8, 112.5, 37.5, 36.9, 25.9, 25.9, 20.3, 17.8. HRMS (*m/z*): (M + H)^+^ (calculated for C_10_H_19_, 139.1481)

**Compound 2c:** was obtained as yellow oil. IR (film): νmax 3500, 2972, 2941, 1715, 1372, 1189, 1027, 973, 911 cm^−1^; 1H NMR (400 MHz, CDCl_3_) δ_H_ 6.33 (d, J = 15.9 Hz, 1H), 5.72–5.55 (m, 2H), 4.97–4.88 (m, 4H), 4.19–4.08 (m, 2H), 3.90 (d, J = 4.0 Hz, 1H), 2.56 (s, 2H), 2.11–2.02 (m, 1H), 1.82 (s, 3H), 1.64–1.27 (m, 5H), 1.23 (t, J = 7.1 Hz, 3H), 0.98 (d, J = 6.7 Hz, 3H); 13C NMR (100 MHz, CDCl_3_) δ_C_ 172.8, 144.5, 141.3, 133.8, 133.7, 131.7, 131.7, 116.8, 113.0, 113.0, 73.5, 73.4, 60.8, 44.6, 44.5, 39.3, 39.3, 38.2, 38.1, 30.3, 30.2, 20.5, 20.4, 18.8, 14.3; HRMS (*m/z*): (M + Na)^+^ calculated for C_16_H_26_O_3_Na: 289.1779, found: 289.1776.

**Compound 2d**: was obtained as a colorless oil; [α] ^22.5^_D_ = 10.9° (c = 0.01 in CHCl_3_); IR (film): νmax 2914, 2856, 1737, 1444, 1374, 1235, 1179, 1146, 1037 cm^−1^ NMR data Table S2 Supplementary Material. HRMS (m/z): (M + Na)^+^ calculated for C_16_H_26_NaO_2_: 273.1825, found: 273.1820.

**Compound 2e**: a colorless oil; [α] ^22^ _D_= −33.0° (c = 0.006 in CHCl3); IR (KBr): νmax 2925, 2871, 1742, 1456, 1377, 1213, 1200, 1151, 1127, 1104, 1034, 999, 957, 944, 883 cm^−1^; NMR data Table S3 Supplementary Material. HRMS (*m/z*): (M + Na)^+^ calculated for C_14_H_20_NaO_5_: 291.1203, found: 291.1207.

### 4.3. Molecular Docking

Molecular docking was performed by Dr. Moussa Sehailia and Dr. Smain Chemat at the Research Centre in Physical and Chemical Analysis (C.R.A.P.C). The method is as follows: the PDB file of SARS-CoV-2 S protein RBD-hACE2 complex (PDB Ref. 6LZG, version 1.0) was obtained from the Research Collaboratory for Structural Bioinformatics (RCSB) protein data bank (PDB) (http://www.rcsb.org/structure/6LZG, accessed on 8 September 2021). UCSF Chimera1.14 was used to visualize the structure of the ligand and/or protein-complex structure, to perform the various functions associated with ligand and protein preparations and acting as an interface to enable molecular docking calculations used locally hosted AutoDock Vina software [16,17] (Pettersen et al., 2004; Trott & Olson, 2010). In addition, all non-standard residues, including that of water, were also removed. The structure of the ligand was incorporated into UCSF Chimera using SMILES string followed by structure minimization. The PDBQT files of the S protein, RBD and the ligand were generated after adding all hydrogens and charges to the structure. The number of binding modes was set to 10 with exhaustiveness of search set to 8. The maximum energy difference was set to 3 kcal mol^−1^. The best scoring pose of the molecule was analyzed in terms of its interaction with the receptor binding motif (RBM). The obtained molecular docking results were then aligned with the PDBQT file of the S protein RBD-hACE2 complex in order to visualize the type of interactions of the docked molecule in the S protein-hACE2 binding interface.

### 4.4. Biological Evaluation—Apoptosis Induction

Apoptosis induction in the presence of compound **2** was carried out as follows. W2 (apoptosis competent) and D3 (apoptosis defective) cells were plated in 96-well plates and incubated for 24 h in growth medium DMEM, after which they were evenly spread at about 50% confluency. At this time, compounds dissolved in DMSO and diluted in growth medium (DMEM) were added to the cells at various concentrations. DMSO concentration was kept at 0.5% in all wells. Plates were incubated for 48 h. Cell viability was determined using a modification of the MTT assay, where the reduction of yellow tertazolium salt (MTT-3-(4,5-Dimethylthiazol-2-yl)-2,5) to purple formazan indicates mitochondrial activity, and thus cell viability. Cells were incubated with 0.5 mg/mL MTT for 3 h. The supernatant was aspirated and DMSO was added to dissolve the formazan crystals. After 30 min incubation at 37˚C with shaking, absorbance was read at 570 nm on a Spectra MAX 250 (Molecular Devices) plate reader. Differential growth from time 0 to 48 h was calculated. Starurosporine, an apoptosis inducer, and DMSO were used as positive and negative controls, respectively.

**HMEC viability rate**: MTT assay was performed to assess the in vitro cell viability rate of normal human mammary epithelial cell line HMEC. Briefly, cells were collected at exponentially growing phases and seeded into 96-well plates (8 × 103 cells/well) in 200 μL of RMPI-1604 culture medium. DMSO (0.01%) was taken as a blank control. Seeded cells were then incubated with varying doses of (−)-6-epi- artemisinin (**2**) (0, 5, 10, 15 and 25 μM) for 48 h. Following (−)-6-epi- artemisinin (**2**) treatment, each well was supplemented with MTT solution (10 μL) and further incubated for about 3 h. Finally, absorbance was recorded at 560 and 630 nm with an Envision microplate reader (PerkinElmer, Waltham, MA, United States) for optical density calculation.

Independent experiments were repeated three times for each assessment and data are presented as mean ± standard deviation *p* < 0.05. Untreated cells were considered as 100% viable cells.

### 4.5. Biological Evaluation—SARS-CoV-2 Inhibition

**Cells and Virus.** Vero E6 cells (ATCC no. 1586) were grown and maintained in minimum Eagle’s medium (Gibco Invitrogen) and supplemented with 10% fetal bovine serum (Gibco Invitrogen) at 37 °C in 5% CO_2_. The SARS-CoV-2 strain (nCoV-2019BetaCoV/Wuhan/WIV04/2019) was propagated, stored and titrated [6]. **Cytotoxicity and Antiviral Assays**. Cytotoxicity was evaluated in Vero E6 cells using a cell counting kit-8 (CCK8) (Beyotime, China) according to the manufacturer’s instructions. For the antiviral assay, 4.8 × 10^6^ Vero E6 cells were seeded onto 48-well cell-culture Petri dishes and grown overnight. After pretreatment with a gradient of diluted experimental compounds for 1 h at 37 °C, cells were infected with the virus at an MOI of 0.01 for 1 h. After incubation, the inoculum was removed, cells were washed with PBS and culture vessels were replenished with fresh drug-containing medium. At 24 h post infection, total RNA was extracted from the supernatant and qRT-PCR was performed to quantify the virus yield [6]. Results are representative of n = 6 and are shown as mean ± SEM. EC_50_ and CC_50_ for each compound were calculated by a four-parameter nonlinear regression model and were plotted by Excel graph.

### 4.6. Biological Evaluation—Antimalaria Assay

100 microliters of a 0.5 mM solution of haematin, previously dissolved in 0.1 M NaOH, was distributed in 96-well U-bottomed microplates. Fifty microliters of (−)-6-epi-artemisinin (**2**) and chloroquine were added to triplicate test wells. Either 50 μL of water or 50 μL of the solvent used to solubilize the drugs was added to control wells. Haematin polymerization was initiated by adding 50μL glacial acetic acid pH of 2.6, and the suspension was incubated at 37 °C for 24 h to allow complete polymerization. Plates were then centrifuged at 8000× *g* rpm for 15 min and the soluble fraction of unprecipitated material was collected (fraction I). The remaining pellet was resuspended with 200 μL of DMSO to remove unreacted haematin. Plates were then centrifuged again at 8000× *g* rpm for 15 min. The DMSO-soluble fraction (fraction II) was collected, and the pellet, consisting of a pure precipitate of haematin, was dissolved in 0.1 M NaOH (fraction III) for spectroscopic quantification. A 150 μL aliquot of each fraction was transferred on to a new plate, and serial four-fold dilutions in 0.1 M NaOH were performed. The amount of haematin was determined by measuring the absorbance at 405 nm using a microtiter plate reader. A standard curve for haematin dissolved in 0.1 M NaOH was used to calculate the amount of porphyrin present in each fraction.

**Statistical analysis.** All experiments were conducted in triplicate measurements and presented as the mean ± SD. Data were analyzed by Excel Microsoft. The IC_50_ values were calculated from nonlinear regression analysis.

## 5. Conclusions

It was demonstrated by this work that the genus *Artemisia* is not the only source of artemisinin (**1**) and analogues. The plant *Saldinia proboscidea* can biosynthesize artemisinin analogue such as (−)-6-epi-artemisinin (**2**); its structure was elucidated by a combination of MS and NMR methods followed by chemical synthesis. According to all biological assay results of (−)-6-epi-artemisinin (**2**), antiproliferative, anti-SARS-CoV-2 and antimalaria, the change in configuration was not critical to artemisinin’s biological properties. Even though the antimalaria and antiproliferative activities of artemisins are important, this research contributes to the anti-SARSCoV-2 potential of artemisinin analogues and provides promising lead candidates for anti-SARS-CoV-2 drug research and development.

## Figures and Tables

**Table 1:** NMR Spectroscopic Data of 6-epi-(−)-artemisinin (**2**) ^1^H (400 MHz, CDCl_3_) and ^13^C (100 MHz, CDCl_3_.

Position	δ_C_, Type	δ_H_, (*J* in Hz)	HMBC ^1^
3	106.8, qC		
4α	37.3, CH_2_	2.41, m	3, 5, 12
4β		2.09, m	5a
5α	24.8, CH_2_	1.90, m	5a, 6
5β		1.10, m	4, 3
5a	51.4, CH	1.38, m	6, 12, 15
6	38.9, CH	1.42, m	5a, 7, 15
7α	34.3, CH_2_	1.10, m	6
7β		1.81, m	8
8α	26.6, CH_2_	2.00, m	7, 8a
8β		2.00, m	7
8a	46.4, CH	1.80, m	8, 9
9	35.0, CH	3.41, dq (7.2, 5.3)	10, 12, 12a
10	173.4, qC		
12	95.1, CH	5.89, s	3, 10, 12a
12a	80.9, qC		
14	26.2, CH_3_	1.46, s	4, 12
15	21.2, CH_3_	1.01, d (5.96)	5a, 6, 7
16	13.9, CH_3_	1.22, d (7.28)	9, 8a, 10

^1^ HMBC correlations, optimized for 8 Hz, are from the proton (s) stated to the indicated carbon.

## Data Availability

The data presented in this study are available in this article.

## Supplementary Materials

The following are available online, ^1^H NMR, ^13^C NMR, COSY, multiplicity edited HSQC, HMBC, ROESY of (−)-6-epi-artemisinin (**2**) and ^1^H NMR, ^13^C NMR of **3**. Figure S1 400 MHz ^1^H NMR spectrum of (−)-6-epi-artemisinin (**2**) in CDCl_3._ Figure S2 100 MHz ^13^C NMR spectrum of (−)-6-epi-artemisinin (2) in CDCl_3._ Figure S3 400 MHz COSY spectrum of (−)-6-epi-artemisinin (**2**) in CDCl_3._ Figure S4 400 MHz Multiplicity edited HSQC spectrum of (−)-6-epi-artemisinin (**2**) in CDCl_3_. Figure S5 400 MHz HMBC spectrum (optimized for J = 8Hz) of (−)-6-epi-artemisinin (**2**) in CDCl_3._ Figure S6 400 MHz ROESY spectrum of (−)-6-epi-artemisinin (**2**) in CDCl_3._ Figure S7 HRESI MS of (**2**). Figure S8 400 MHz ^1^H NMR spectrum of synthetic compound (**2**) in CDCl_3._ Figure S9 100 MHz ^13^C NMR spectrum of synthetic compound (**2**) in CDCl_3._ Figure S10 400 MHz ^1^H NMR spectrum of mixture of natural (**2**) and synthetic (**2**) in CDCl_3._ Figure S11 100 MHz ^13^C NMR spectrum of mixture of natural (**2**) and synthetic (2) in CDCl_3._ Figure S12 400 MHz ^1^H NMR spectrum of compound (**3**) in CDCl_3_. Figure S13 100 MHz ^13^C NMR spectrum of compound (**3**) in CDCl_3_. Figure S14 Taxonomic comparison between the species *Saldinia proboscidea* and *Artemisia annua L* Table S1 ^1^H (400 MHz, CDCl_3_) and ^13^C (100 MHz, CDCl_3_) NMR Data of (+)-artemisinin (**1**), (−)-artemisinin and the 9 epimer of 2 (**3**). Table S2. NMR Spectroscopic Data of compound (**2d**) 1H (400 MHz, CDCl_3_) and ^13^C (100 MHz, CDC_l3_). Table S3. NMR Spectroscopic Data of compound (**2e**) ^1^H (400 MHz, CDCl_3_) and ^13^C (100 MHz, CDCl_3_). Detail synthesis steps.

## Sample Availability

Samples of the compounds are not available from the authors.
