# Supplementary material for: (−)-6-epi-Artemisinin, a Natural Stereoisomer of (+)-Artemisinin in the Opposite Enantiomeric Series, from the Endemic Madagascar Plant Saldinia proboscidea, an Atypical Source
Source: Molecules. 2021 Sep 12;26(18):5540. doi: 10.3390/molecules26185540 (PMC8472513; doi:10.3390/molecules26185540)
Supplement: Supplementary file 1 [file molecules-26-05540-s001.zip › molecules-1346763-supplementary.pdf]

Supplementary Material

(-)-6-epi-Artemisinin, a Natural Stereoisomer of (+)-Artemisinin in the Opposite Enantiomeric Series, from the Endemic Madagascar Plant *Saldinia proboscidea*, an Atypical Source

Saholinirina Marie Hortensia Randrianarivo[†], Claudine Aimée Rasolohery[†], Sitraka Mitombina Rafanomezantsoa[†], Razafindrakoto Heriniaina Randriamampionona[†], Liti Haramaty[§], Roger Marie Rafanomezantsoa[†] and Eric H. Andrianasolo [‡⊥*]

* To whom correspondence should be addressed Tel.: (519) 893-2213

E-mail: eric.andrianasolo@uwaterloo.ca

[†]Université de Fianarantsoa - Ecole doctorale de Géochimie et Chimie Médicinale (GEOCHIMED) Fianarantsoa Madagascar

[‡]Centre National de Recherches Industrielle et Technologique (CNRIT) Tananarive Madagascar

[§]DMCS, Rutgers, The State University of New Jersey

[⊥]University of Waterloo, Biology Department, 200 University Ave W, Waterloo, Ontario N2L3G1

Figure S1. 400 MHz ^{1}H NMR spectrum of (-)-6-epi-artemisinin (**2**) in $CDCl_3$
Figure S2. 100 MHz ^{13}C NMR spectrum of (-)-6-epi-artemisinin (**2**) in $CDCl_3$
Figure S3 400 MHz COSY spectrum of (-)-6-epi-artemisinin (**2**) in $CDCl_3$
Figure S4 400 MHz Multiplicity edited HSQC spectrum of (-)-6-epi-artemisinin (**2**) in $CDCl_3$
Figure S5 400 MHz HMBC spectrum (optimized for *J* = 8Hz) of (-)-6-epi-artemisinin (**2**) in $CDCl_3$
Figure S6 400 MHz ROESY spectrum of (-)-6-epi-artemisinin (**2**) in $CDCl_3$
Figure S7 HRESI MS of (**2**)
Figure S8 400 MHz ^{1}H NMR spectrum of synthetic compound (**2**) in $CDCl_3$
Figure S9 100 MHz ^{13}C NMR spectrum of synthetic compound (**2**) in $CDCl_3$
Figure S10 400 MHz ^{1}H NMR spectrum of mixture of natural (**2**) and synthetic (**2**) in $CDCl_3$
Figure S11 100 MHz ^{13}C NMR spectrum of mixture of natural (**2**) and synthetic (**2**) in $CDCl_3$
Figure S12 400 MHz ^{1}H NMR spectrum of compound (**3**) in $CDCl_3$
Figure S13 100 MHz ^{13}C NMR spectrum of compound (**3**) in $CDCl_3$
Figure S14 Taxonomic comparison between the species *Saldinia proboscidea* and *Artemisia annua L*
Table S1 ^{1}H (400 MHz, $CDCl_3$) and ^{13}C (100 MHz, $CDCl_3$) NMR Data of (+)-artemisinin (**1**)[6]**,** (-)-artemisinin[5] and the 9 epimer of **2** (**3**)
Table S2. NMR Spectroscopic Data of compound (**2d**) ^{1}H (400 MHz, $CDCl_3$) and ^{13}C (100 MHz, $CDCl_3$)
Table S3. NMR Spectroscopic Data of compound (**2e**) ^{1}H (400 MHz, $CDCl_3$) and ^{13}C (100 MHz, $CDCl_3$)
Detail synthesis steps

Figure S1. 400 MHz ^{1}H NMR spectrum of (-)-6-epi-artemisinin (**2**) in $CDCl_3$

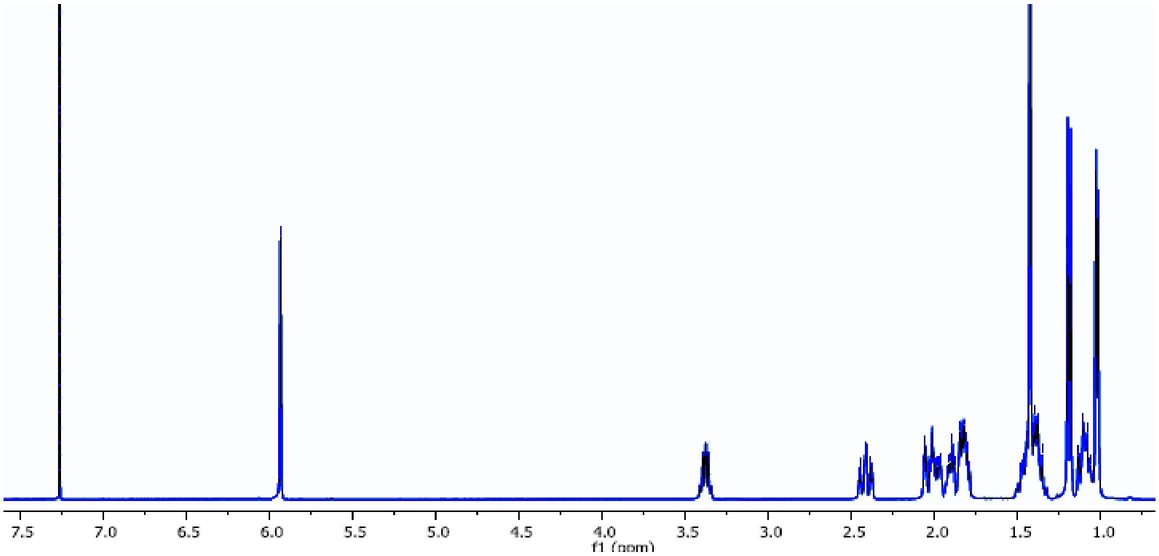

Figure S2. 100 MHz ^{13}C NMR spectrum of (-)-6-epi-artemisinin (**2**) in $CDCl_3$

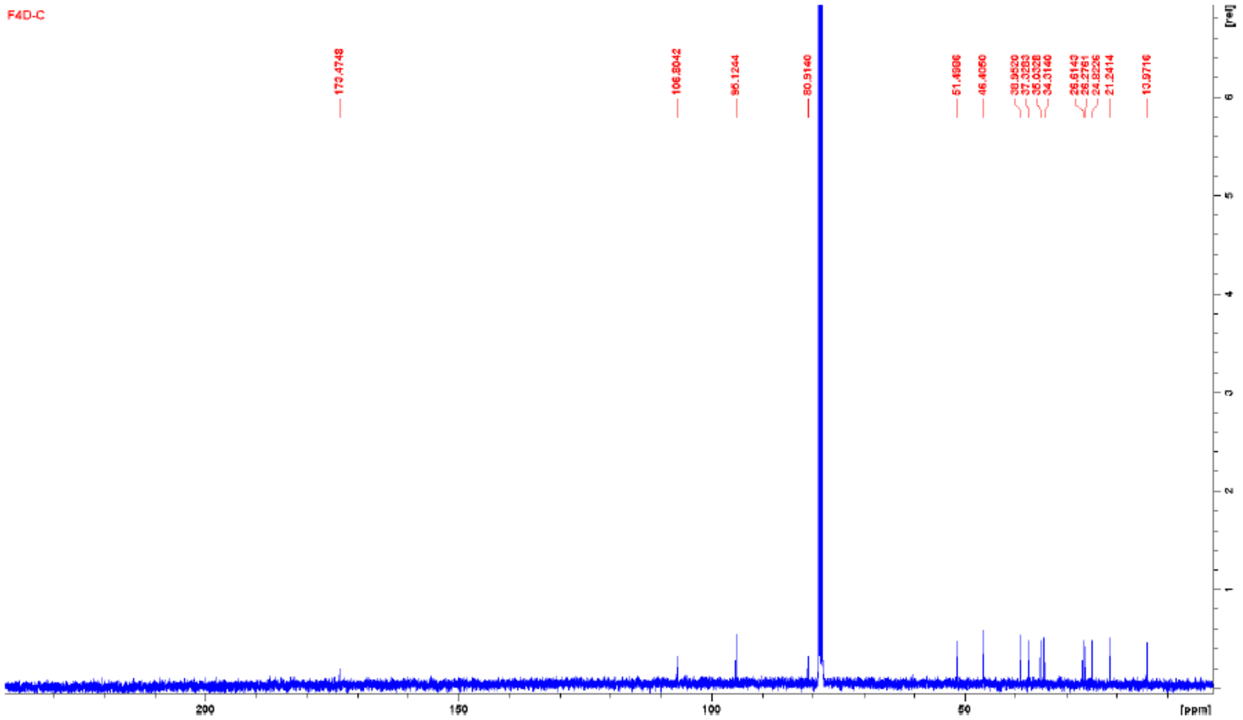

Figure S3 400 MHz COSY spectrum of (-)-6-epi-artemisinin (**2**) in $CDCl_3$

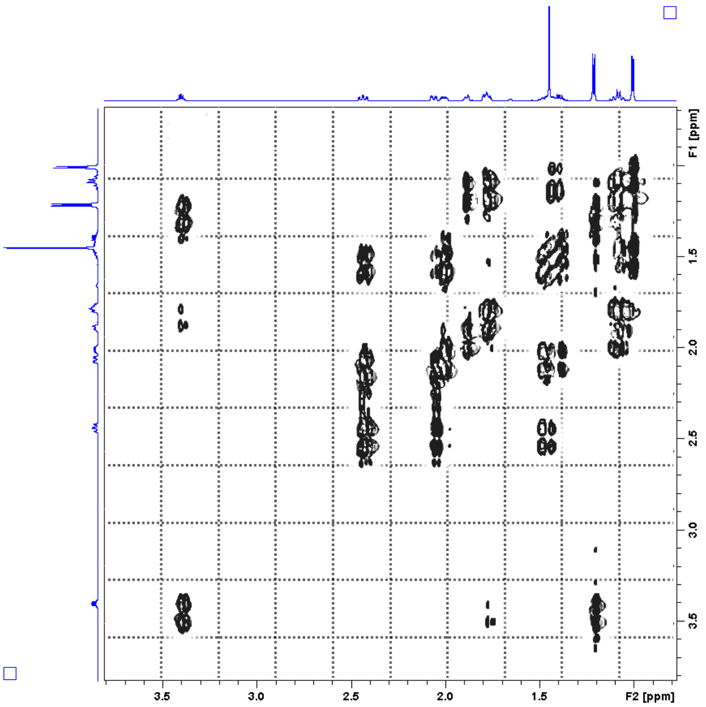

Figure S4 400 MHz Multiplicity edited HSQC spectrum of (-)-6-epi-artemisinin (**2**) in $CDCl_3$

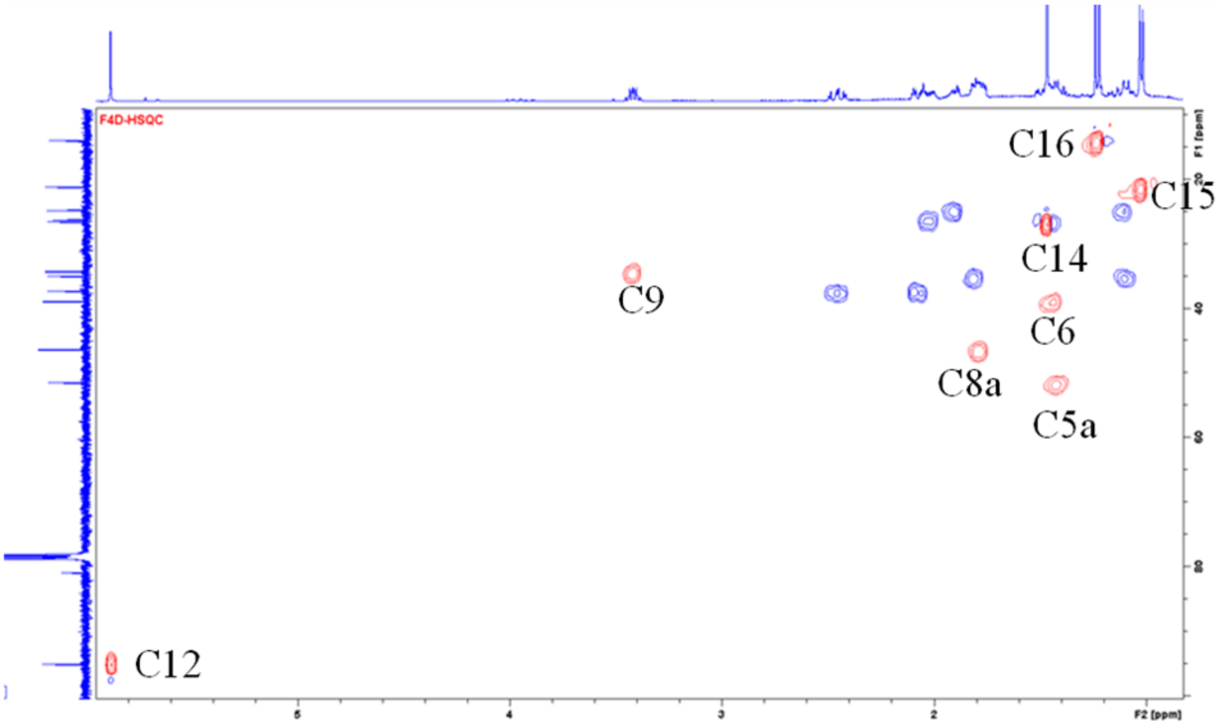

Figure S5 400 MHz HMBC spectrum (optimized for *J* = 8Hz) of (-)-6-epi-artemisinin (**2**) in $CDCl_3$

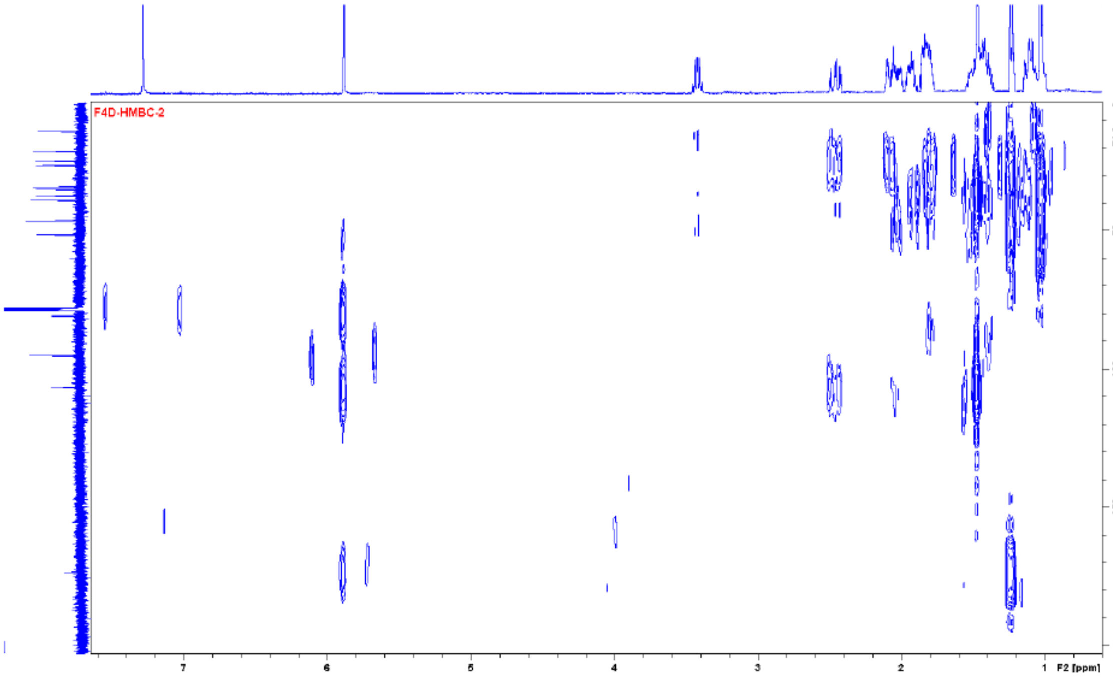

Figure S6 400 MHz ROESY spectrum of (-)-6-epi-artemisinin (**2**) in $CDCl_3$

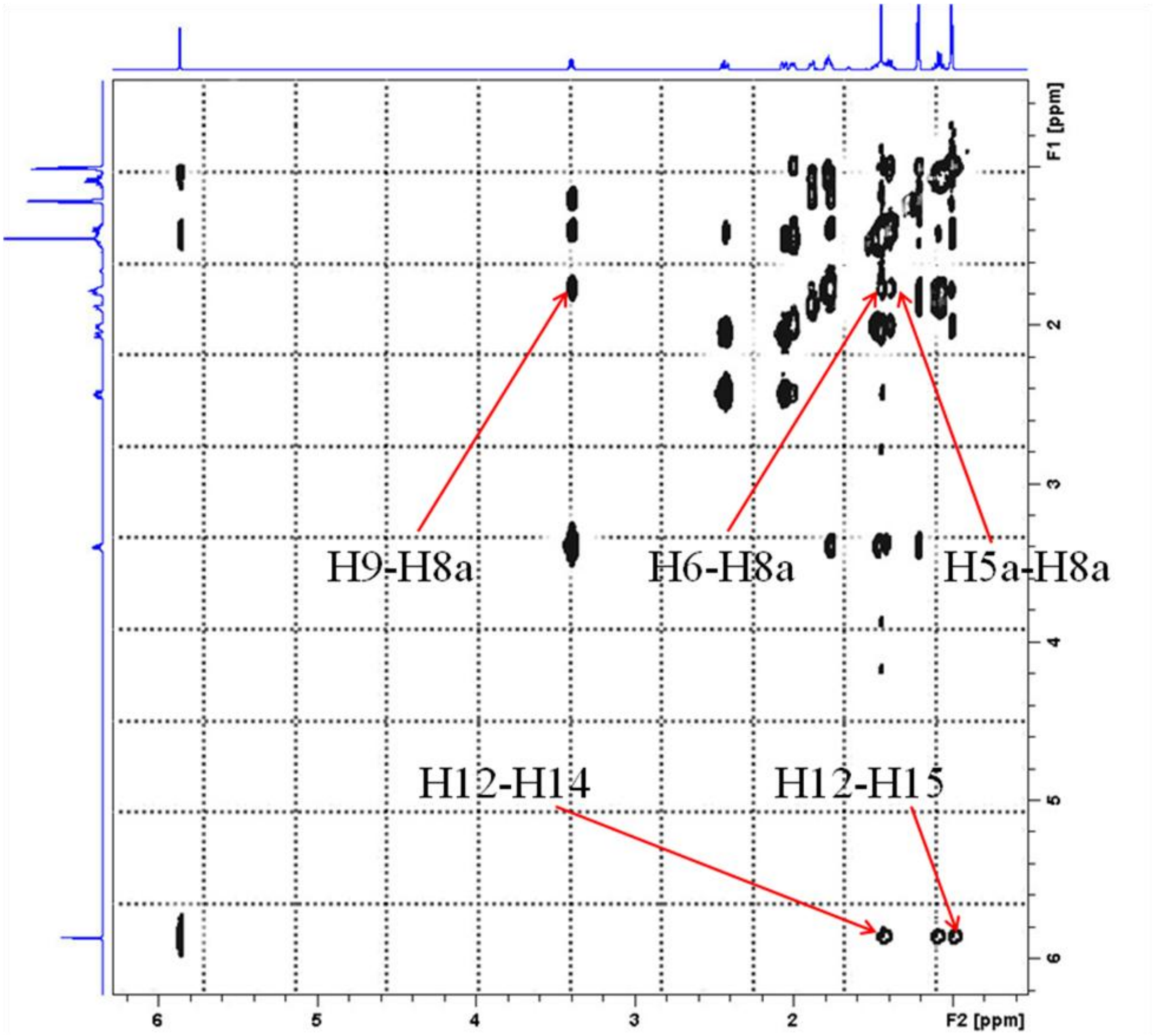

Figure S7 HRESI MS of (**2**)

m/z **283.1547** $(M+H)^+$ m/z **265.2123** $(M-H_2O+H)+$ m/z **247.1990** $(M-2H_2O+H)+$

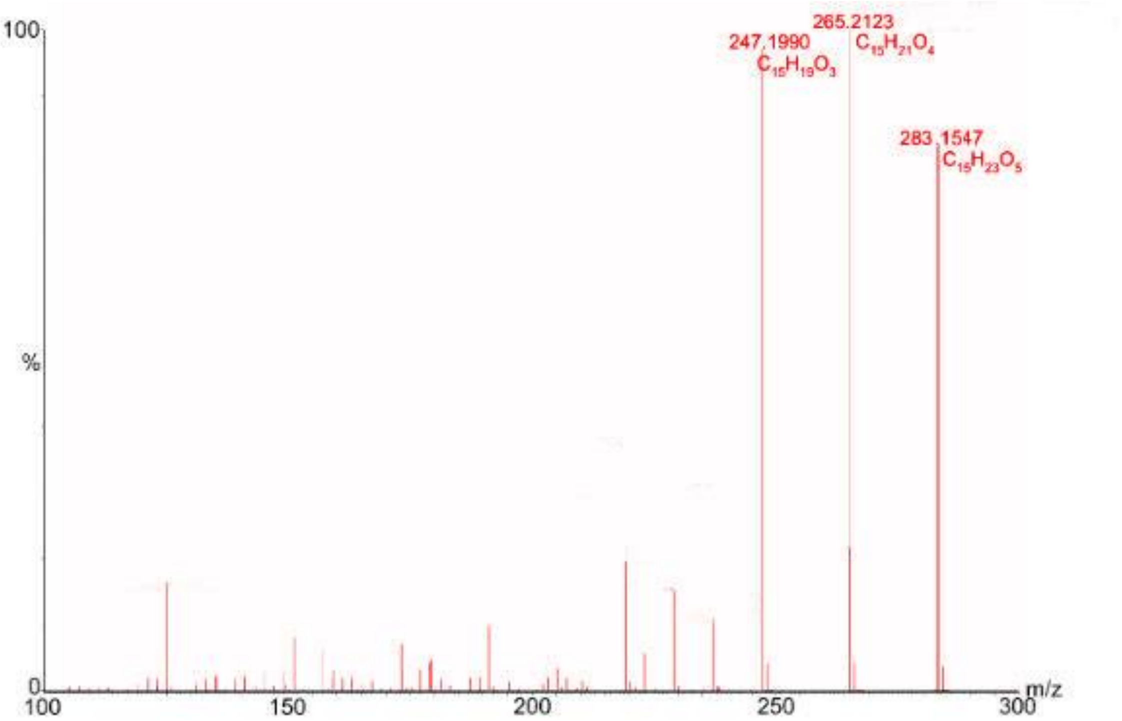

Figure S8 400 MHz ^{1}H NMR spectrum of synthetic compound (**2**) in $CDCl_3$

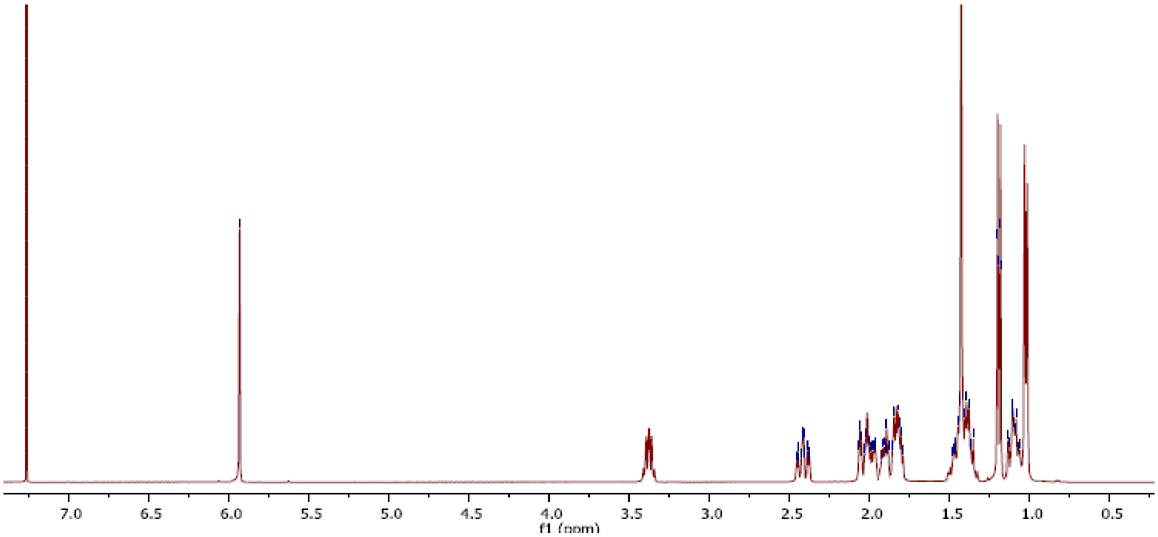

Figure S9 100 MHz ^{13}C NMR spectrum of synthetic compound (**2**) in $CDCl_3$

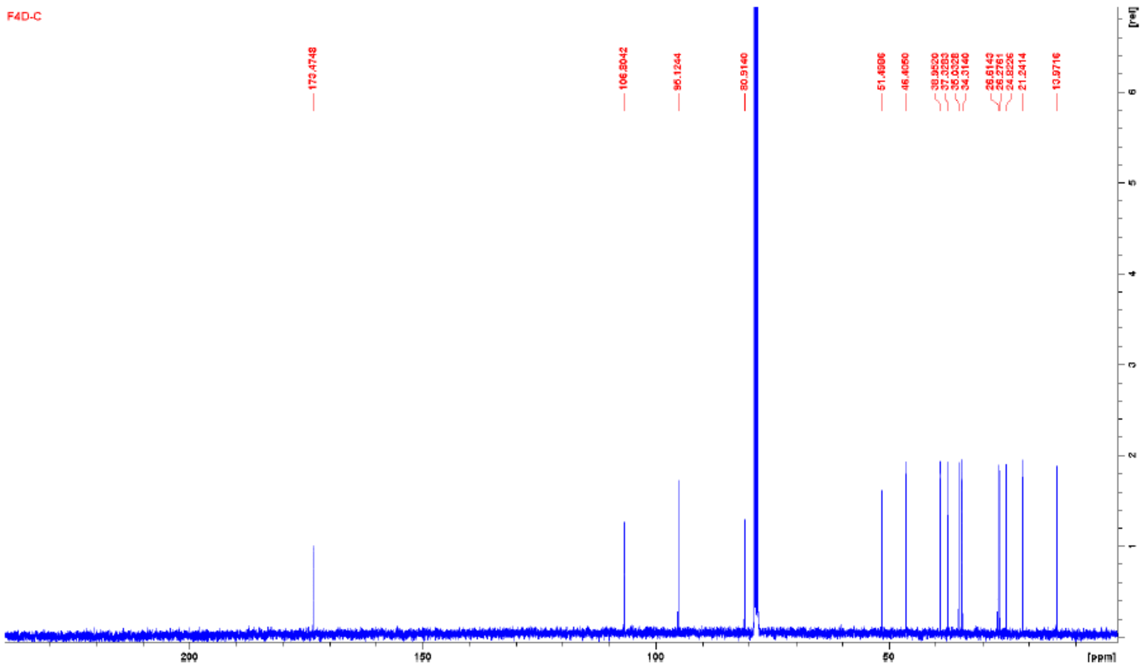

Figure S10 400 MHz ^{1}H NMR spectrum of mixture of natural (**2**) and synthetic (**2**) in $CDCl_3$

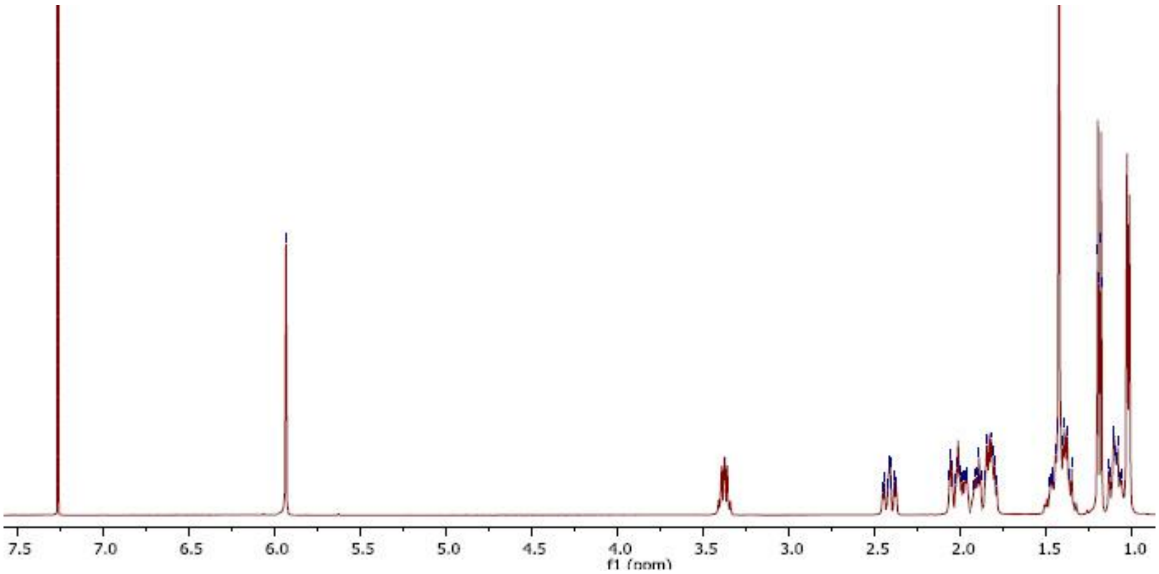

Figure S11 100 MHz ^{13}C NMR spectrum of mixture of natural (**2**) and synthetic (**2**) in $CDCl_3$

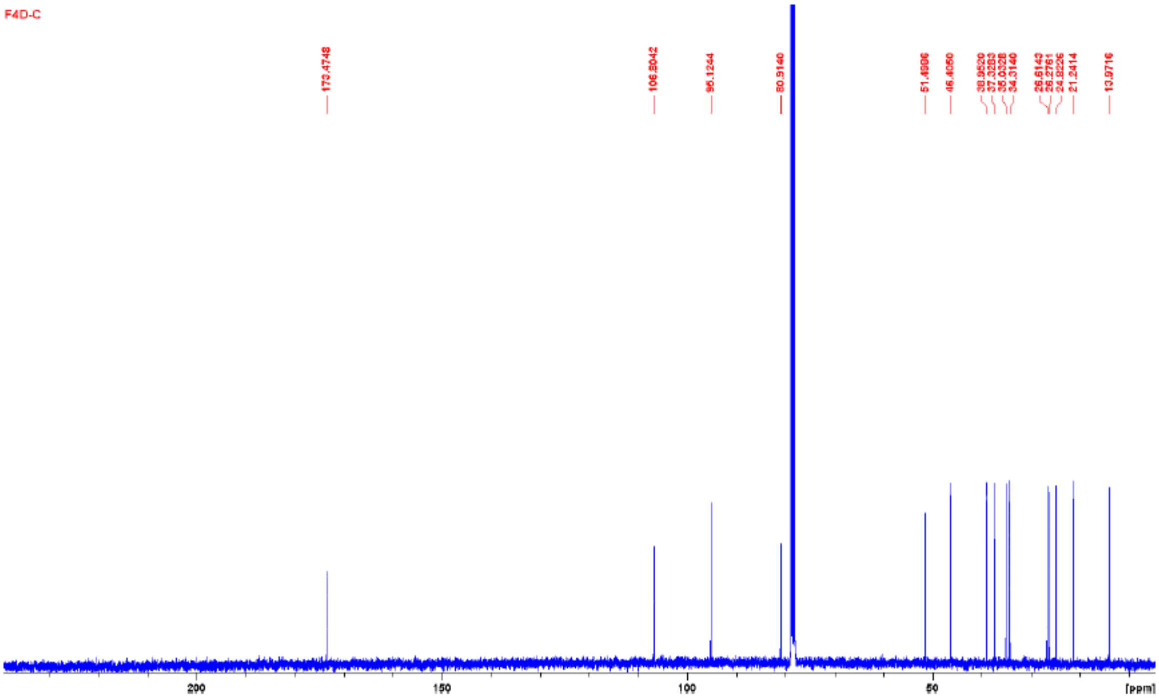

Figure S12 400 MHz ^{1}H NMR spectrum of compound (**3**) in $CDCl_3$

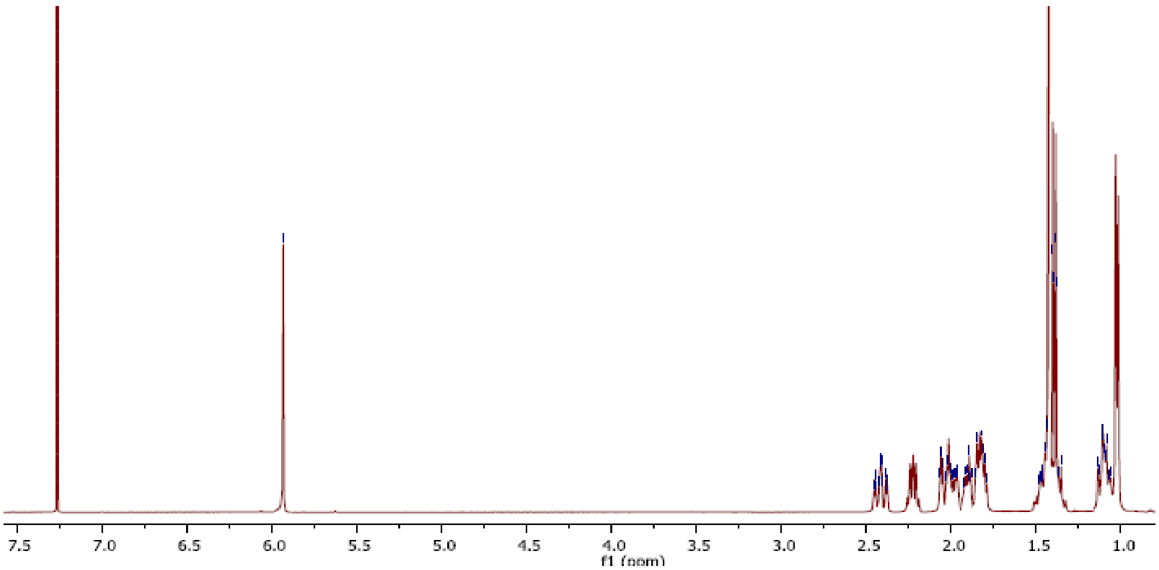

Figure S13 100 MHz ^{13}C NMR spectrum of compound (**3**) in $CDCl_3$

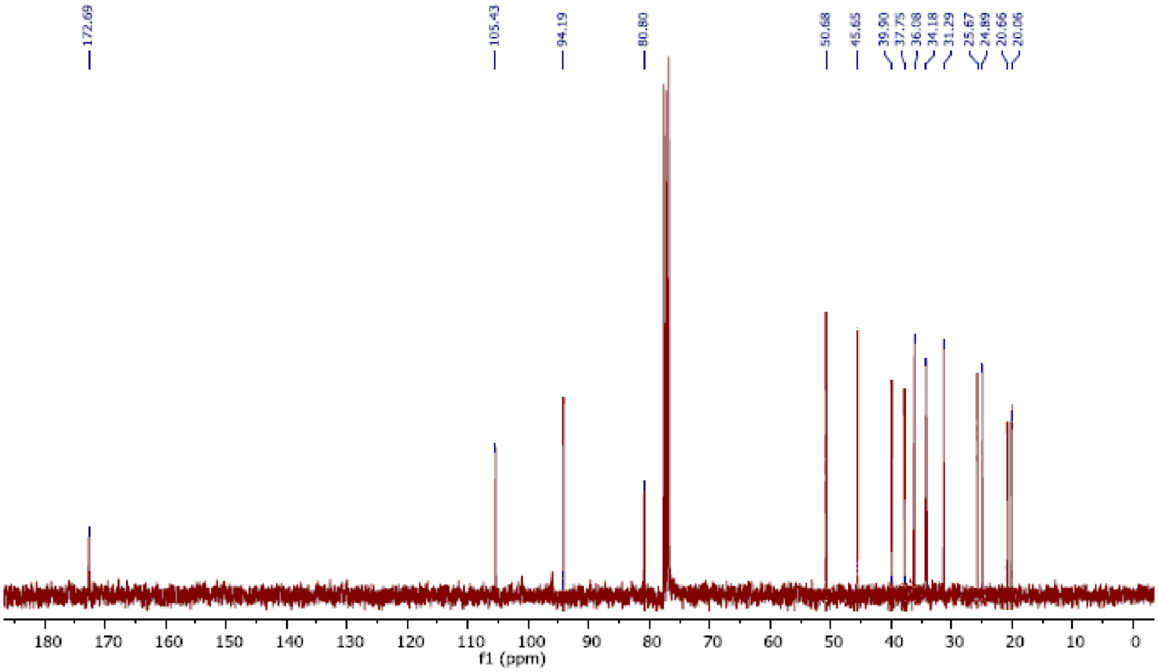

Figure S14 Taxonomic comparison between the species *Saldinia proboscidea* and *Artemisia annua L*

Saldinia proboscidea

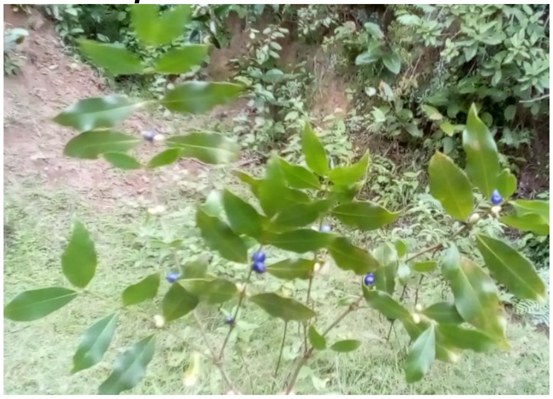

Kingdom: *Plantae*
Phylum : *Tracheophyta*
Class: *Magnoliopsida*
Order: *Gentianales*
Family: *Rubiaceae*
Genus: *Saldinia*
Species: *Saldinia proboscidea*

***Artemisia annua L*.**

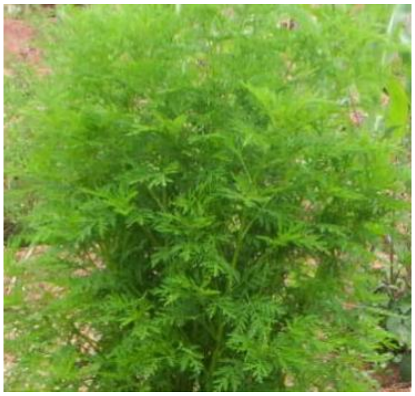

Kingdom: *Plantae*
Subkingdom: *Tracheobionta*
Superdivision: *Spermatophyta*
Division: *Magnoliophyta*
Class: *Magnoliopsida*
Subclass: *Asteridae*
Order: *Asterales*
Family: *Asteraceae*
Genus: *L*.
Species: *annua L.*

Table S1. ^{1}H (400 MHz, $CDCl_3$) and ^{13}C (100 MHz, $CDCl_3$) NMR Data of (+)-artemisinin (**1**)[6], (-)-artemisinin[5] and the 9 epimer of **2** (**3**)

	(+)-artemisinin (**1**)		(-)-artemisinin		9 epimer of **2** (**3**)	
position	δ_C	$\delta_{H,}$ (J in Hz)[a]	δ_C	$\delta_{H,}$ (J in Hz)	δ_C	$\delta_{H,}$ (J in Hz)
3	105.2		105.5		105.4	
4α	35.7	2.43, ddd (14.7, 13.6, 4.2)	36.0	2.47, m	36.1	2.39, m
4β		2.05, ddd (14.7, 5.62, 3.4)		2.08, m		2.06, m
5 α	24.7	2.01, m (14.5, 5.5)	25.0	1.94, m	24.9	1.90, m
5 β		1.47, m (14.5, 5.5)		1.48, m		1.10, m
5a	49.9	1.37, m (11.5, 7.0)	50.1	1.48-1.34, m	50.7	1.38, m
6	37.4	1.42, m (13.0, 6.4)	37.6	1.48-1.34, m	37.8	1.42, m
7 α	33.4	1.08, m (13.5)	33.7	1.71, m	34.2	1.10, m
7 β		1.79, m (13.5)		1.79, m		1.81, m
8 α	23.3	1.87, m (14.0)	23.5	1.90, m	31.3	2.00, m
8 β		1.12, m (14.0)		1.79. m		2.00, m
8 a	44.8	1.75, m (13.5, 5.5)	45.1	1.79-1.71, m	45.7	1,78, m
9	32.7	3.40, dq (7.2, 5.4)	33.0	3.42-3.33, m	39.9	2.27, dq (7.2, 5.3)
10	171.9		172.2		172.7	
12	93.6	5.87, s	93.8	5.84, s	94.2	5.89, s
12a	79.3		79.6		80.8	
14	25.1	1.44, s	25.3	1.48, s	25.7	1.42, s
15	19.7	0.99, d (6)	20.0	0.98, d (5.8)	20.1	1.01, d (5.9)
16	12.4	1.21, d (7.3)	12.7	1.22-1.16, d (7.4)	20.7	1.37, d (7.28)

a Multiplicity and coupling constants (Hz) are shown in parentheses. For overlapped multiplets, not every coupling constant could be identified, and in some cases only the half-height of the signal (in Hz) could be determined.

Table S2. NMR Spectroscopic Data of compound (**2d**) ^{1}H (400 MHz, $CDCl_3$) and ^{13}C (100 MHz, $CDCl_3$)

Compound **2d**		
Position	δ_C, type	δ_H, (*J* in Hz)
1	173.8, qC	
2	39.2, CH_2	2.68, dd (14.6, 3.7) 2.00, m
3	60.3, CH_2	4.13, q (7.1)
4	14.5, CH_3	1.26, t (7.1)
1'	39.1, CH	1.54, m
2'	33.1, CH_2	1.76, m 1.12, m
3'	35.8, CH	1.60, m 1.01, m
4'	37.5, CH	1.36, m
4a'	47.5, CH	1.42, m
5'	26.7, CH_2	1.99, m 1.10, m
6'α	31.0, CH_2	1.95, m
7'	135.1, qC	
8'	122.6, CH	5.38, m
8a'	45.9, CH	1.47, m
9'	21.0, CH_3	1.01, d (5.9)
10'	23.9, CH_3	1.65, s

Table S3. NMR Spectroscopic Data of compound (**2e**) ^{1}H (400 MHz, $CDCl_3$) and ^{13}C (100 MHz, $CDCl_3$)

	Compound **2e**	
position	δ_C, type	δ_H, (*J* in Hz)
3	105.6, qC	
4α	36.0, CH_2	2.48 , m
4β		2.35, m
5 α	24.9, CH_2	2.10, m
5 β		1.82, m
5a	50.2, CH	1.50, m
6	37.6, CH_2	1.42, m
7 α	34.0, CH_2	1.08, m (13.5)
7 β		1.79, m (13.5)
8 α	29.5, CH_2	1.78, m (14.0)
8 β		1.19, m (14.0)
8 a	38.9, CH	1.82, m
9	31.7, CH_2	3.18, dd (18.2, 6.9) 2.27, dd (18.2, 1.2)
10	168.9, qC	
12	93.9, CH	5.91, s
12a	78.6, qC	
14	25.5, CH_3	1.44, s
15	21.1, CH_3	1.01, d (5.8 Hz)

Detail synthesis steps

Compound 2b To a stirred solution of (-)-Citronellol (36.7 g, 235 mmol; *TCI Europe, > 99 % ee*) in THF (400 mL) was added NaH (14.1 g, 353 mmol, 60% in mineral oil) at 0°C. After stirring for 5 h at rt, CS2 (16.8 mL, 21.1 g, 277 mmol) was added slowly and it was stirred overnight. Methyliodide (18.4 mL, 41.7 g, 294 mmol) was added, followed by stirring for three more hours. Water (400 mL) and hexane (300 mL) was added, the phases were separated and the aqueous phase was extracted with hexane (2 x 300 mL). The combined organic phases were washed with water and dried over Na2SO4. The solvent was removed under reduced pressure and the crude product was purified by silica gel chromatography (*n*-hexane) to yield (*S*)-*O*-(3,7-dimethyloct-6-en-1-yl) *S*-methyl carbonodithioate (88.4 g, 359 mmol) which was warmed to 280°C in a Kugelrohrdestillation-apparatus. Heating was continued till no more fragmentation product was produced and the crude product was destilled under vacuum again (95 mbar, 90 °C → 160 °C) delivering (-)-Citronellene (37.8 g, 273 mmol, 76 %) as a yellow liquid. From **Compound 2b** to **ketone 2c':** followed all steps from Krieger et al. [7],

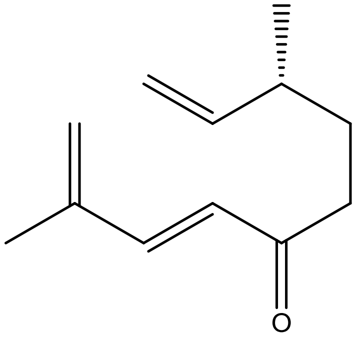

Ketone 2c'

Compound 2c A suspension of activated zinc dust (1.83 g, 28.1 mmol), iodine (141 mg, 556 µmol) and toluene (50 mL) was stirred under reflux for 5 min and cooled to room temperature. To this mixture ethyl bromoacetate (1.87 g, 1.24 mL, 11.1 mmol) was added first. Afterwards, **ketone 2c'** (1.00 g, 5.61 mmol) solved in toluene (10 mL) was added to the suspension. The resulting mixture was stirred at 90 °C for 30 min. The reaction was cooled to 0 °C and water (20 mL) was added. The suspension was filtered and the filtrate was extracted with MTBE (3 x 50 mL). The combined organic phases were washed with water (50 mL) and saturatedNaCl-solution (50 mL), dried over Na2SO4 and concentrated under reduced pressure. The crude product was purified by silica gel chromatography (*n*-hexane/EtOAc, 15:1 v/v).

Compound 2d: Lithium (10.1 mg, 1.45 mmol) was added in portions to liquid ammonia (3 mL) at −70 °C. The resulting blue suspension was stirred for 30 minutes at this temperature. Afterwards, the mixture was cooled to −76 °C and ethanol (14.7 mg, 18.6 µL, 319 µmol) and alkene from previous step (similar to Krieger et al. [7]) (72.2 mg, 291 µmol) solved in Et2O (1.5 mL) was added. The reaction was stirred 10 min and saturated NH4Cl-solution (2 mL) was added slowly. The mixture was warmed to room temperature and stirred for 3 h. The phases were separated and the aqueous phase was extracted with MTBE (3 x 3 mL). The combined organic phases were washed with water (5 mL) and saturated NaCl-solution (5 mL), dried over Na_2SO_4 and concentrated under reduced pressure. The crude product was purified by silica gel chromatography (*n*hexane/ EtOAc, 30:1 v/v).

Compound 2e: Through a solution of Compound **2d** (45 mg, 0.180 mmol) and methylene blue (~ 5 mg) in DCM (60 mL) was bubbled a continuous stream of O2 (~ 50

mL/min) and the reaction mixture was irradiated with light (150 W) for five hours at −30 °C. All volatile components were removed under reduced pressure (30 °C) and the crude product was filtered through a short plug of celite (*n*-hexane/EtOAc, 3:1 v/v). The crude hydroperoxide was dissolved in DCM (40 mL) under O2-atmosphere and some drops of TFA were added at 0°C. It was warmed to rt overnight and stirred for further two days. Saturated $NaHCO_3$-solution (10 mL) and water was added. The phases were separated and the aqueous phase was extracted with DCM (2 x 50 mL). The combined organic phases were washed with saturated NaCl-solution and dried over Na_2SO_4. The solvent was removed under reduced pressure and the crude product was purified by silica gel chromatography (*n*-hexane/EtOAc, 10:1 → 6:1 v/v) to yield compound **2e**
